# Supplementary material for: In-situ grafting of cobalt phthalocyanine on gas diffusion electrodes enables ampere-level CO2 reduction
Source: Nat Commun. 2025 Dec 8;17:124. doi: 10.1038/s41467-025-66808-3 (PMC12775133; doi:10.1038/s41467-025-66808-3)
Supplement: Supplementary file 1 — Supplemental Information [file 41467_2025_66808_MOESM1_ESM.docx]

**In-situ grafting of cobalt phthalocyanine on gas diffusion electrodes enables ampere-level CO_2_ reduction**

Huihui Yan^1,2^, Gaoxiang He^2,3^, Rongli Fan^1,2^, Minyue Zhao^1,2^, Huiting Huang^1,2^, Zhexing Lin^1,2^, Bin Gao^1,2^, Jianyong Feng^1,2*^, Zhigang Zou^1,2,3^, Zhaosheng Li^1,2*^

^1^National Laboratory of Solid State Microstructures, College of Engineering and Applied Sciences, Nanjing University, 22 Hankou Road, Nanjing 210093, China

^2^Jiangsu Key Laboratory of Nano Technology, Nanjing University, 22 Hankou Road, Nanjing 210093, China

^3^School of Physics, Nanjing University, Nanjing 210093, China.

**Contents**

Supplementary Figures………………………………………………………………………. 3

Supplementary Tables………………………………………………………………………. 24

References…………………………………………………………………………………… 28

**
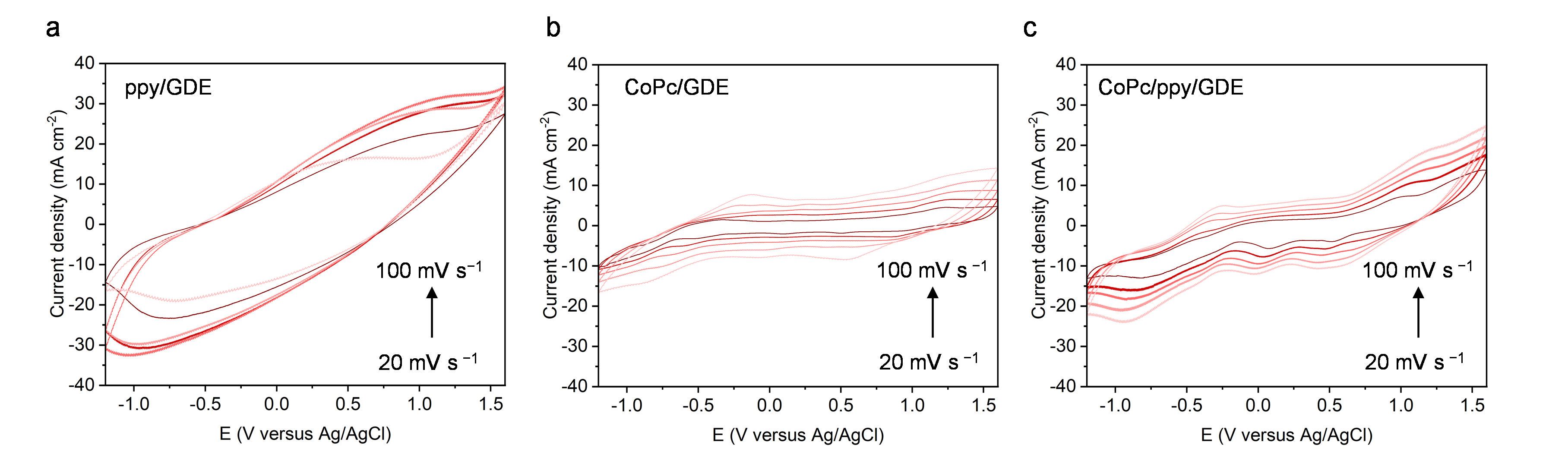
**

**Supplementary Fig. 1.** CV patterns of ppy/GDE (**c**) in 0.01 M 1-butyl-3-methylimidazolium tetrafluoroborate ([BMIM]BF_4_) with 4 mM pyrole, CoPc/GDE (**d**) in 0.01 M [BMIM]BF_4_ with 1 mM CoPc and CoPc/ppy/GDE (**e**) in [BMIM]BF_4_ with 4 mM pyrole and 1 mM CoPc at different scan rates (from 20 to 100 mV s^−1^). All potentials were calibrated to the Ag/AgCl scale and electrochemical measurements were performed at room temperature (23 ± 2℃). The resistance value is 15 ± 2 Ω. There is no iR correction for voltages. Source data are provided as a Source Data file.


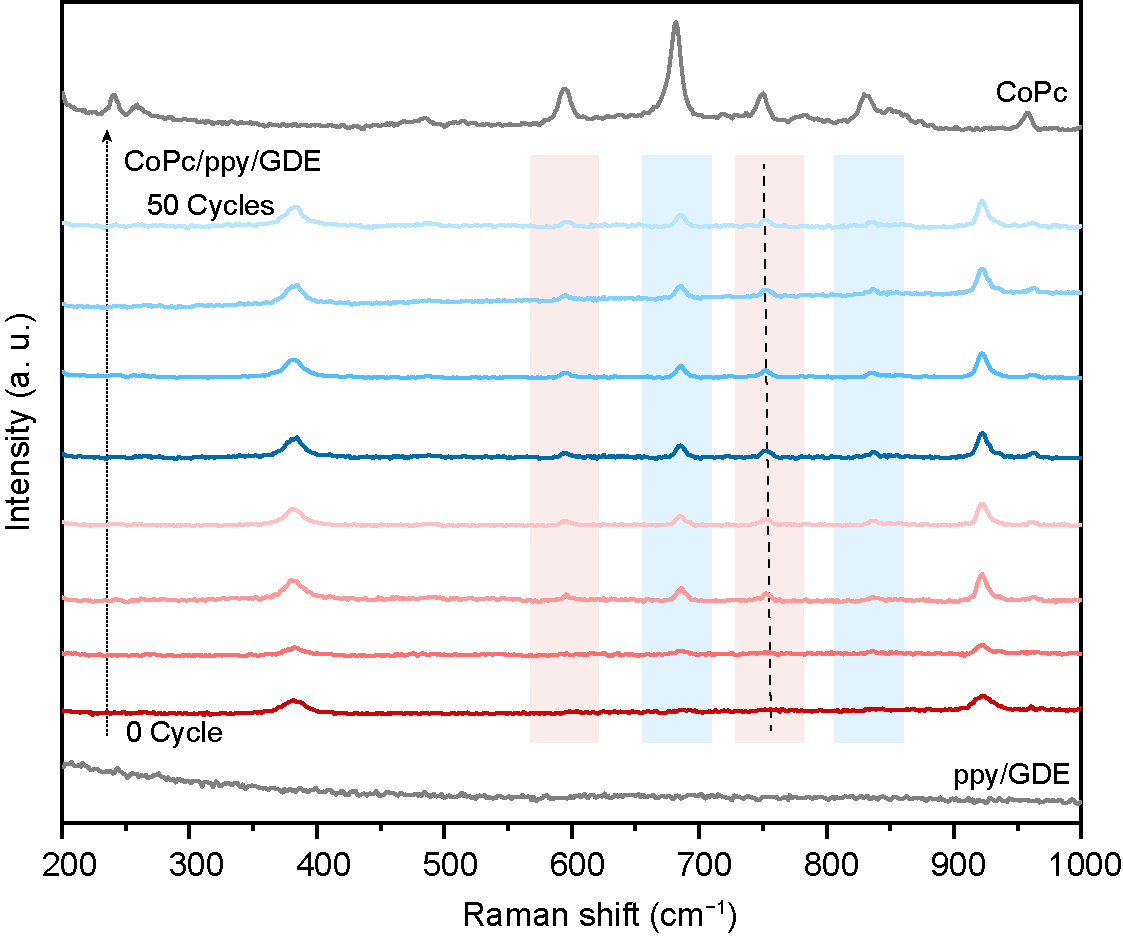


**Supplementary Fig. 2.** In situ Raman spectra of CoPc/ppy/GDE collected at different CV cycles in 0.1 M [BMIM]BF_4_ with 4 mM pyrole and 1 mM CoPc.


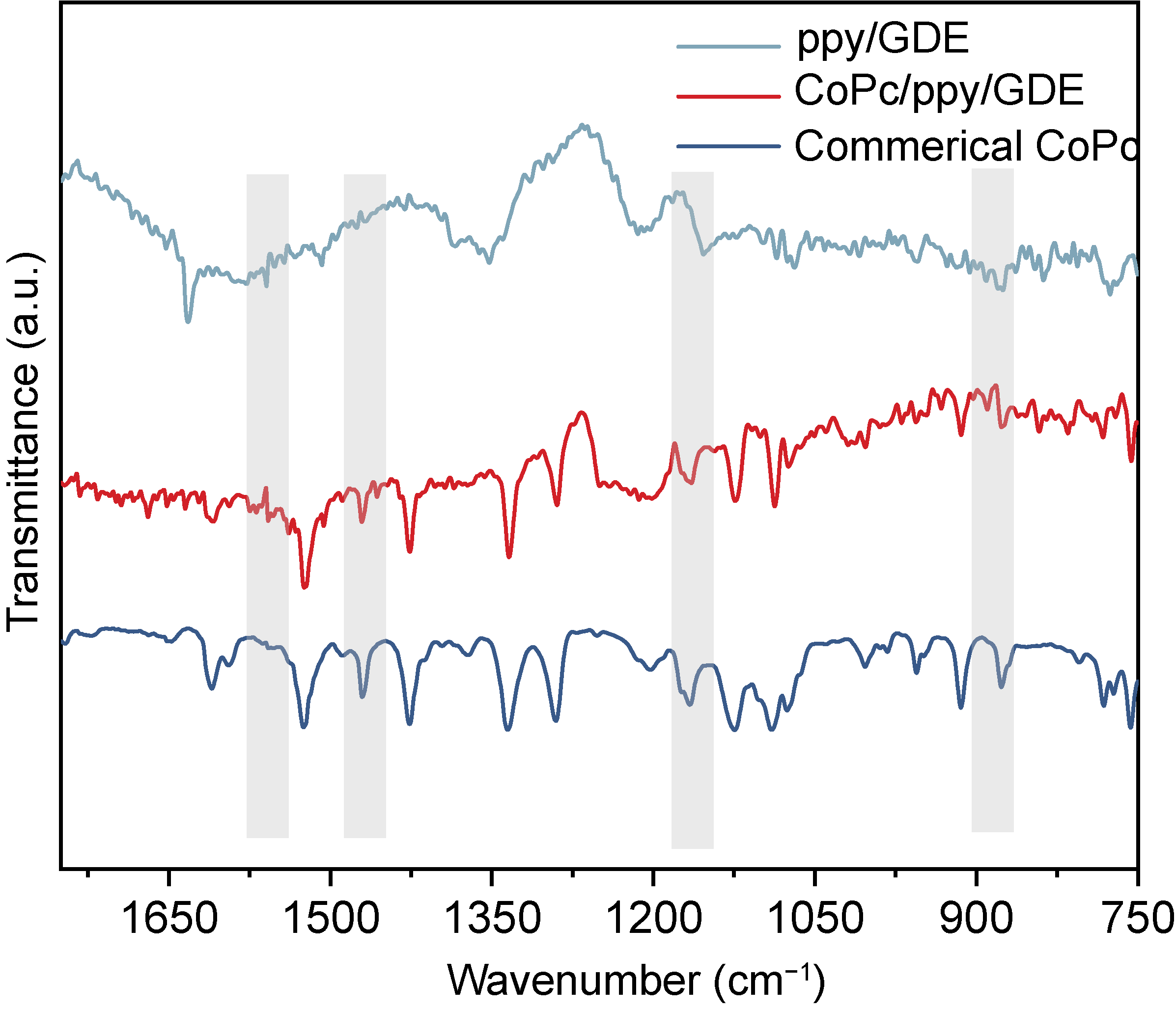


**Supplementary Fig. 3.** FTIR spectra of CoPc/GDE, CoPc/ppy/GDE and ppy/GDE.


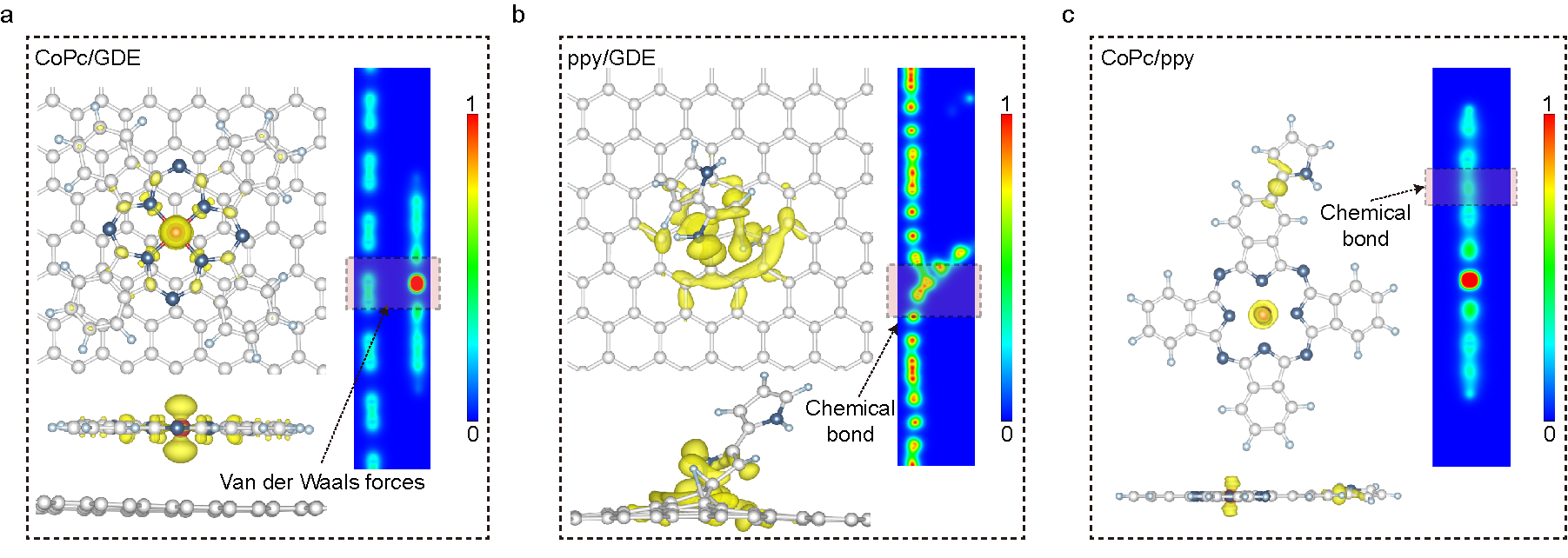


**Supplementary Fig. 4.** Charge density redistribution (left) and electron localization function (right) of CoPc/GDE (**a**), ppy/GDE (**b**) and (**c**) CoPc/ppy. The color code is as follows: gray represents carbon atoms, blue represents nitrogen atoms, and pink represents cobalt atoms. The yellow region shows electron gain.

Differential charge density and electron localization function (ELF) analyses (Supplementary Fig. 4a) reveal weak van der Waals interactions between CoPc and the GDE due to negligible charge accumulation, whereas distinct electron enrichment at the ppy/GDE interface suggests stronger interfacial coupling. Conversely, pronounced electron accumulation and green ELF signatures between ppy and CoPc (Supplementary Figs. 4b and c) confirm the intensive of chemical bond, consistent with their electronic interaction.


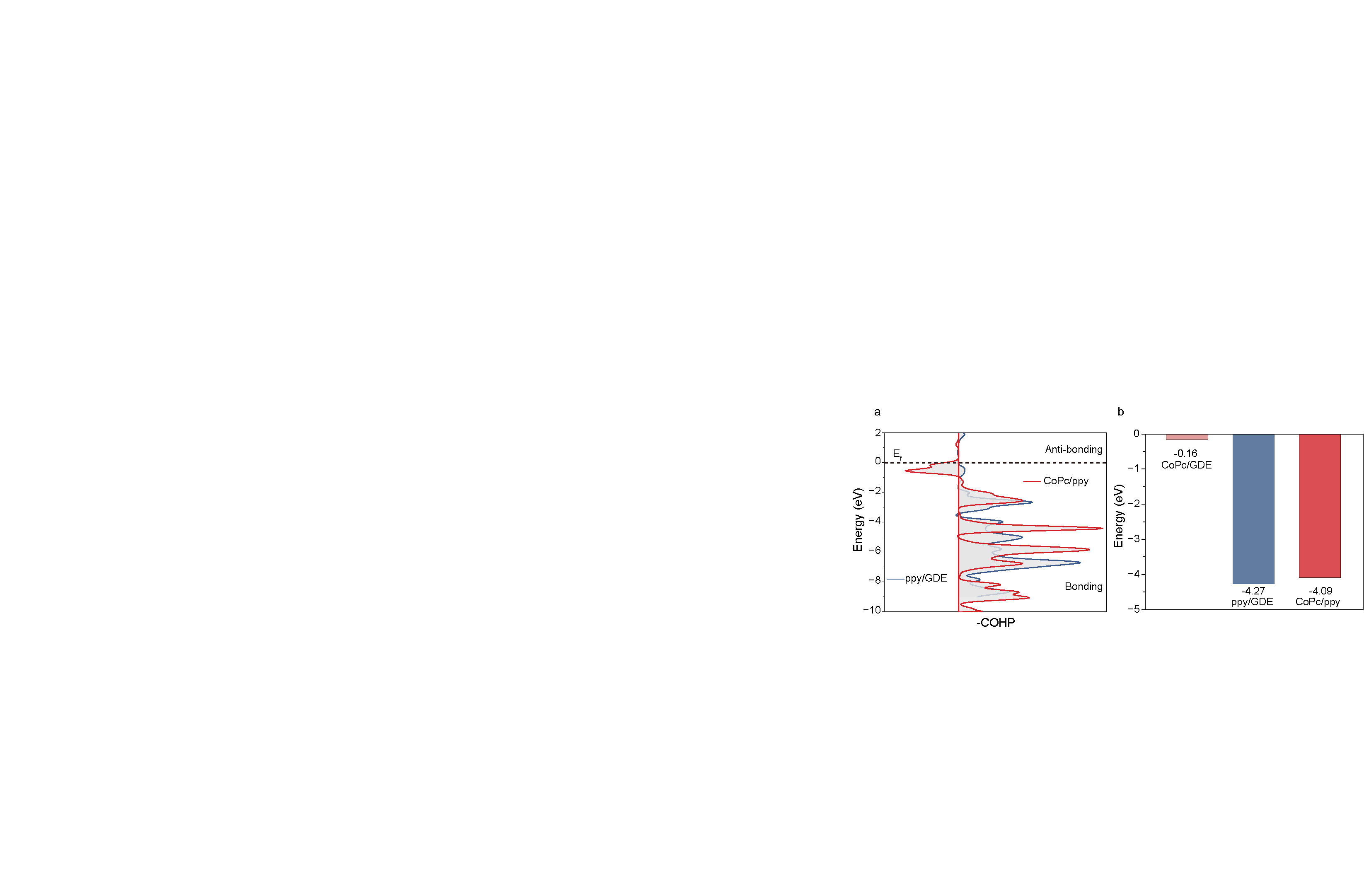


**Supplementary Fig. 5. a**, COHP analysis of the C–C bond between GDE and ppy and ppy/CoPc; note that −COHP is used as the a-axis. **b**, ICOHPs of CoPc/GDE, ppy/GDE and CoPc/ppy.

Crystal orbital Hamilton population (COHP) analysis was employed to calculate the bonding strength of CoPc/GDE, ppy/GDE and CoPc/ppy. As illustrated in Supplementary Fig. 5a, below the Fermi level, the −COHP values of CoPc/ppy and ppy/GDE are positive, indicating that ppy forms chemical bonds with CoPc/ppy and ppy/GDE. To quantitatively represent the bonding strength, the integrated crystal orbital Hamilton population (ICOHP) calculation was conducted (Supplementary Fig. 5b). The values of CoPc/GDE, ppy/GDE and CoPc/ppy are −0.16, −4.27 and −4.09 eV, respectively. These results indicate that the covalent bonding force is more robust than the van der Waals force.


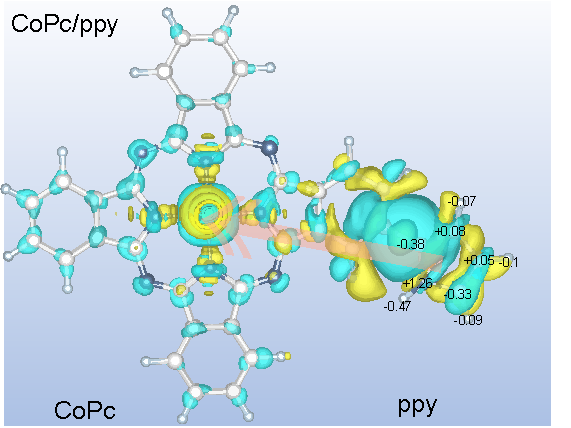


**Supplementary Fig. 6.** Schematic diagram of differential charge density of CoPc/ppy and Bader charge analysis. The cyan (yellow) region shows electron loss (gain).

Bader charge analysis suggests that ppy has a tendency to transfer electrons to CoPc (Supplementary Fig. 6). The ppy acts as a linker to anchor CoPc onto the GDE through covalent bonds, while simultaneously modulating the electronic structure of CoPc for improved catalytic performance.


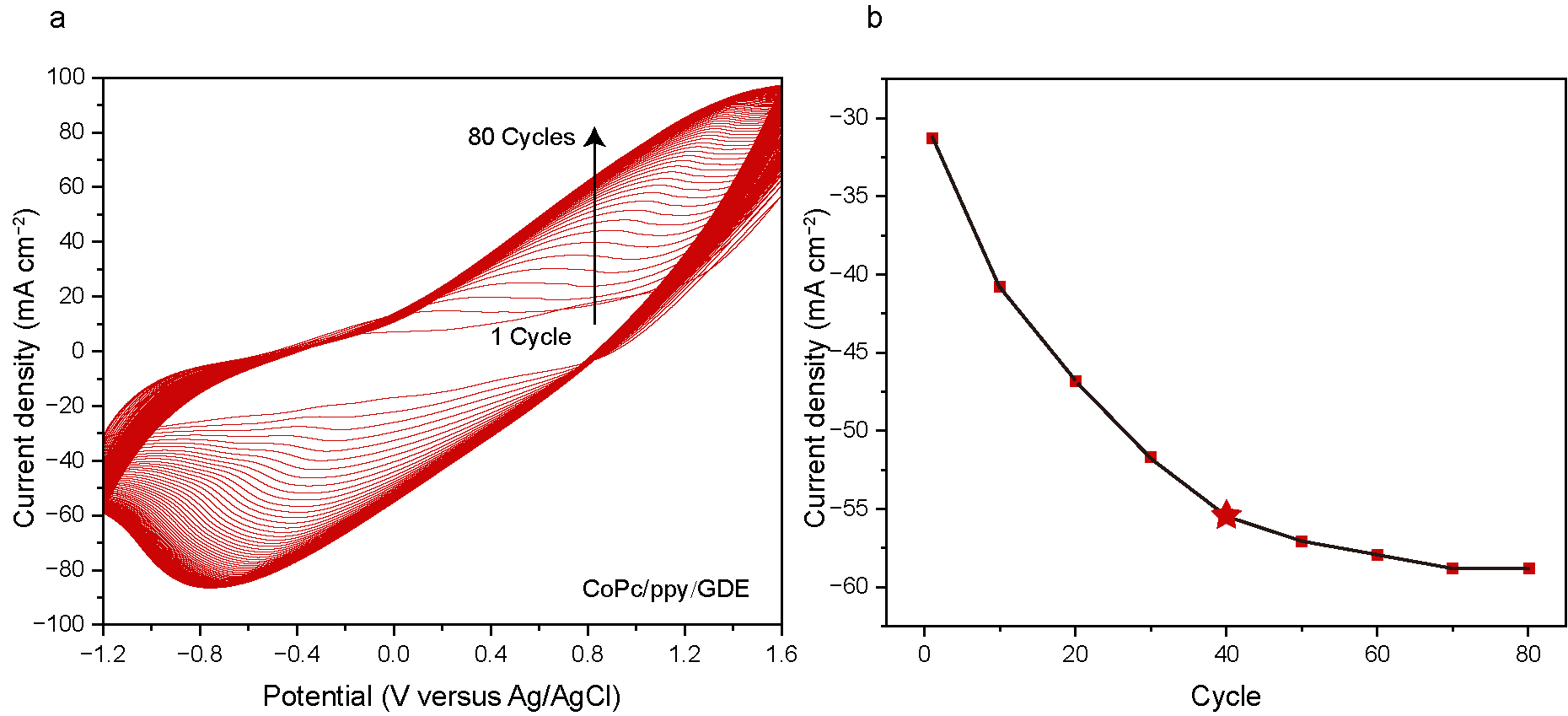


**Supplementary Fig. 7.** **a**, The different deposition cycles of CoPc/ppy/GDE during the electro-polymerization in 0.01 M [BMIM]BF_4_ with 4 mM pyrole and 1 mM CoPc at 100 mV s^−1^. **b**, The variation of current density with the number of deposition cycles. All potentials were calibrated to the Ag/AgCl scale and electrochemical measurements were performed at room temperature (23 ± 2℃). The resistance value is 15 ± 2 Ω. There is no iR correction for voltages. Source data are provided as a Source Data file.

In our approach, precise control over the molecular weight or chain length of ppy is difficult because the electropolymerization process is dynamically influenced by various steps including monomer diffusion, radical coupling, and CoPc incorporation. Despite the lack of precise control over the ppy molecular weight, our CoPc/ppy/GDE catalysts show controllable and reproducible electrochemical behaviors towards CO_2_RR, when the electropolymerization conditions are well organized. Specifically, the electrochemical CO_2_RR performance of CoPc/ppy/GDE electrodes can be systematically and finely optimized through tuning two key parameters including the number of CV scans and the feed ratio of pyrrole to CoPc, based on which the chain length of ppy and CoPc loading/incorporating could be controlled.

Supplementary Fig. 7 illustrates the effects of CV number during electropolymerization, the current density that corresponds to the electrochemical growth of CoPc and ppy on GDE initially increases with cycle number but exhibits a saturation trend beyond 40 cycles. We infer that mass transport or ppy resistance issues gradually dominate and cease the continuous growth of CoPc and ppy on GDE in the applied CV potential range. Correspondingly, the CO_2_RR performance of yielded CoPc/ppy/GDE electrodes begins to stabilize from 40 CV cycles (Supplementary Fig. 8). Beyond 40 CV cycles, the gain in current density becomes limited, along with the consideration of synthesis time, the CV number for CoPc/ppy/GDE fabrication is set at 40.


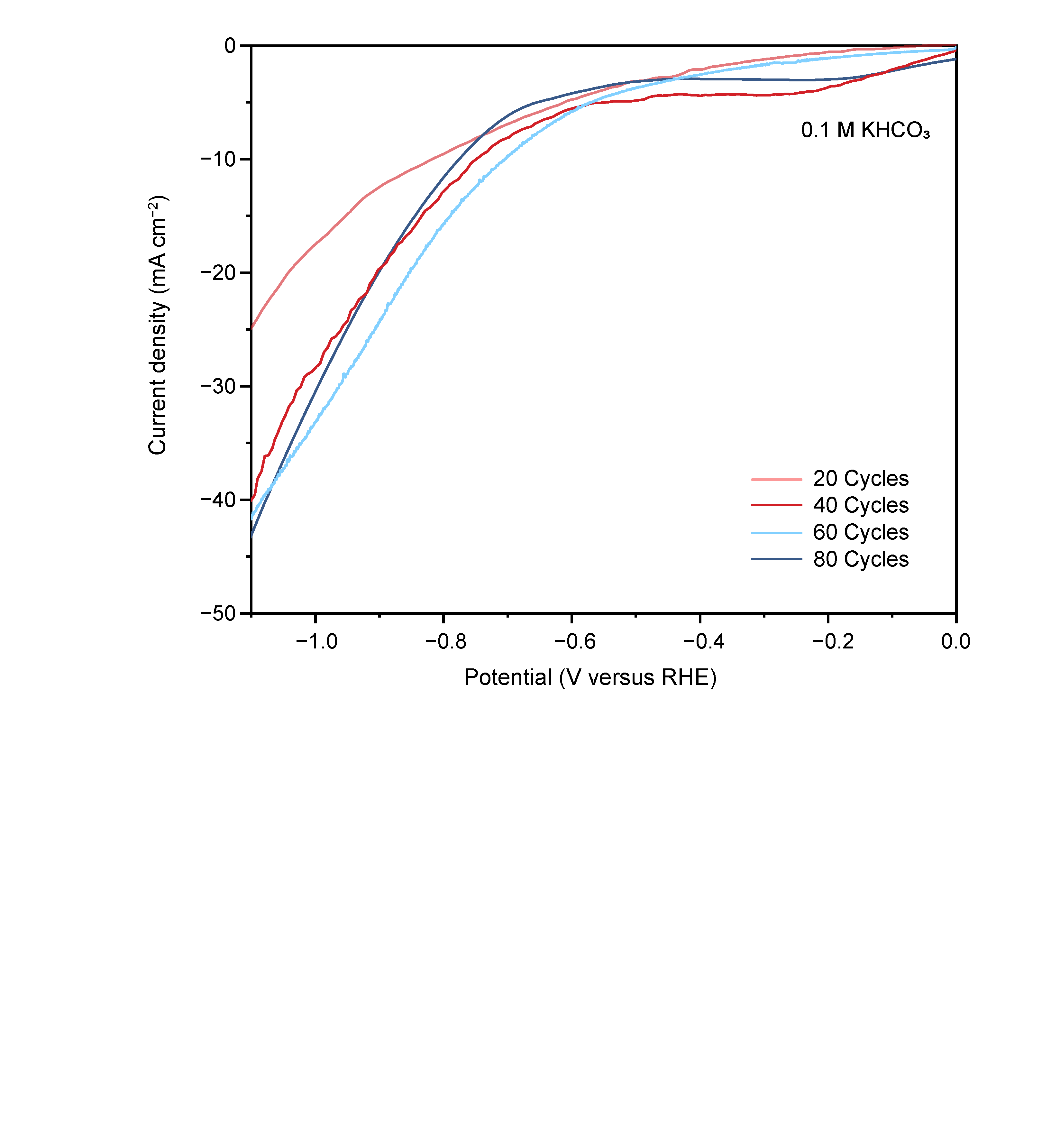


**Supplementary Fig. 8.** The LSV of CoPc/ppy/GDE during CO_2_RR in CO_2_-saturated 0.1 M KHCO_3_ with an H-type cell. The resistance value of H-cell is 10 ± 2 Ω. The gas flow rate of H-cell is 5 mL/min. All electrochemical measurements were performed at room temperature (23 ± 2℃) in 0.1 M CO_2_-saturated KHCO_3_ solution (pH=6.8 ± 0.2). There is no iR correction for voltages. Source data are provided as a Source Data file.


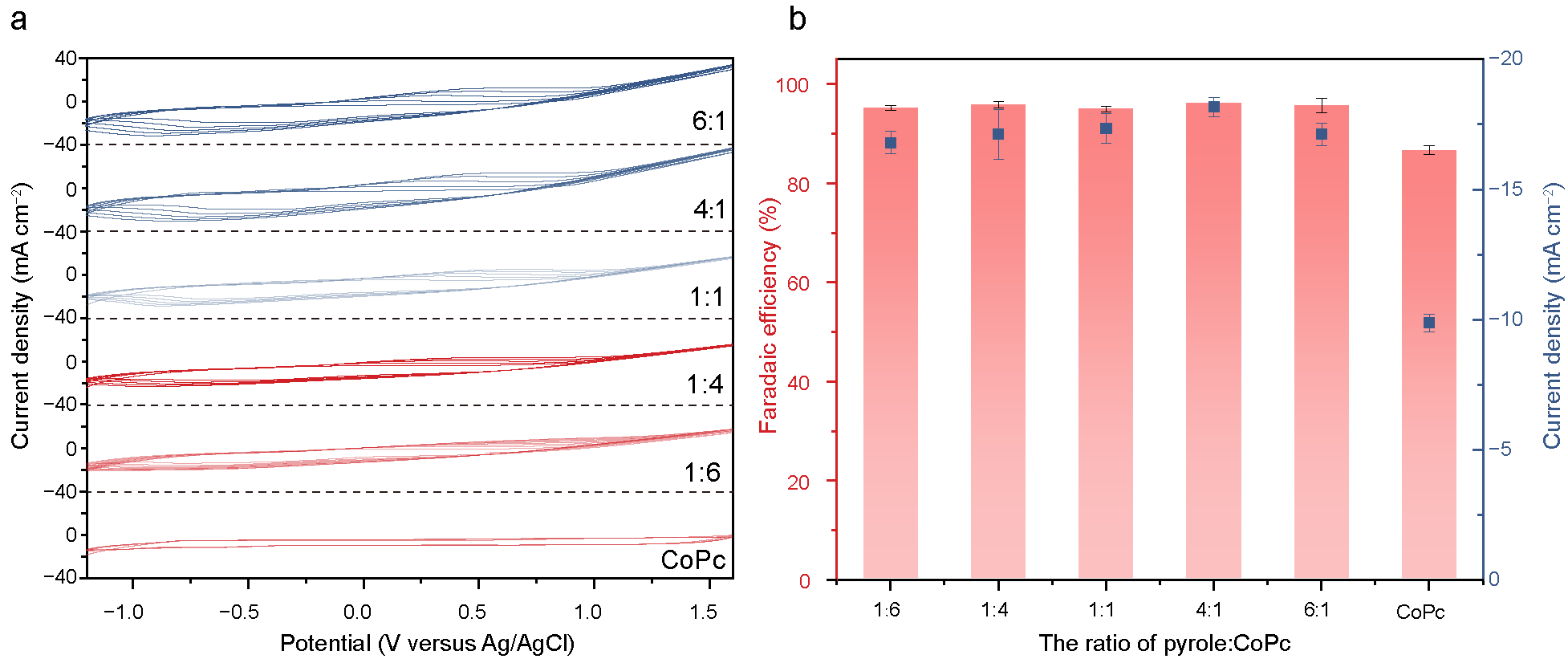


**Supplementary Fig. 9.** **a**, The CV patterns of the growth of CoPc/ppy on GDE in 0.1 M [BMIM]BF_4_ with different ratios of pyrole and CoPc. The potentials were calibrated to the Ag/AgCl scale and electrochemical measurements were performed at room temperature (23 ± 2℃). The resistance value is 15 ± 2 Ω. There is no iR correction for voltages.**b**, The Faradaic efficiency and current density of CoPc/ppy/GDE synthesized by different precursors of pyrole and CoPc at −1.0 V versus RHE during CO_2_RR in CO_2_-saturated 0.1 M KHCO_3_ with an H-type cell (pH=6.8 ± 0.2). There is no iR correction for voltages. Data are presented as mean values ± standard deviation. The standard deviation is obtained based on three independent samples. Source data are provided as a Source Data file.

Considering that the CO_2_RR performance of CoPc/ppy/GDE electrodes is closely correlated with the loading/incorporating amount of CoPc, we optimized the synthetic procedure by varying the pyrrole-to-CoPc feed ratio (1:6 to 6:1). As shown in Supplementary Fig. 9, only small activity differences could be observed for these CoPc/ppy/GDE electrodes obtained in the presence of both pyrrole and CoPc, and the best performing one is obtained with a high pyrrole-to-CoPc ratio of 4:1; when only CoPc is applied, the yielded CoPc/GDE electrode shows largely suppressed CO_2_RR activity. Based on these results, we infer that the electropolymerization step of ppy dominates the whole formation process of CoPc/ppy/GDE electrodes, as pristine CoPc is chemically less active towards electropolymerization; under this condition, ppy initiates the C–C coupling process between GDEs and ppy, ppy and pristine CoPc, thereby realizing covalent grafting of pristine CoPc onto carbon-based GDEs via robust C–C bonds.


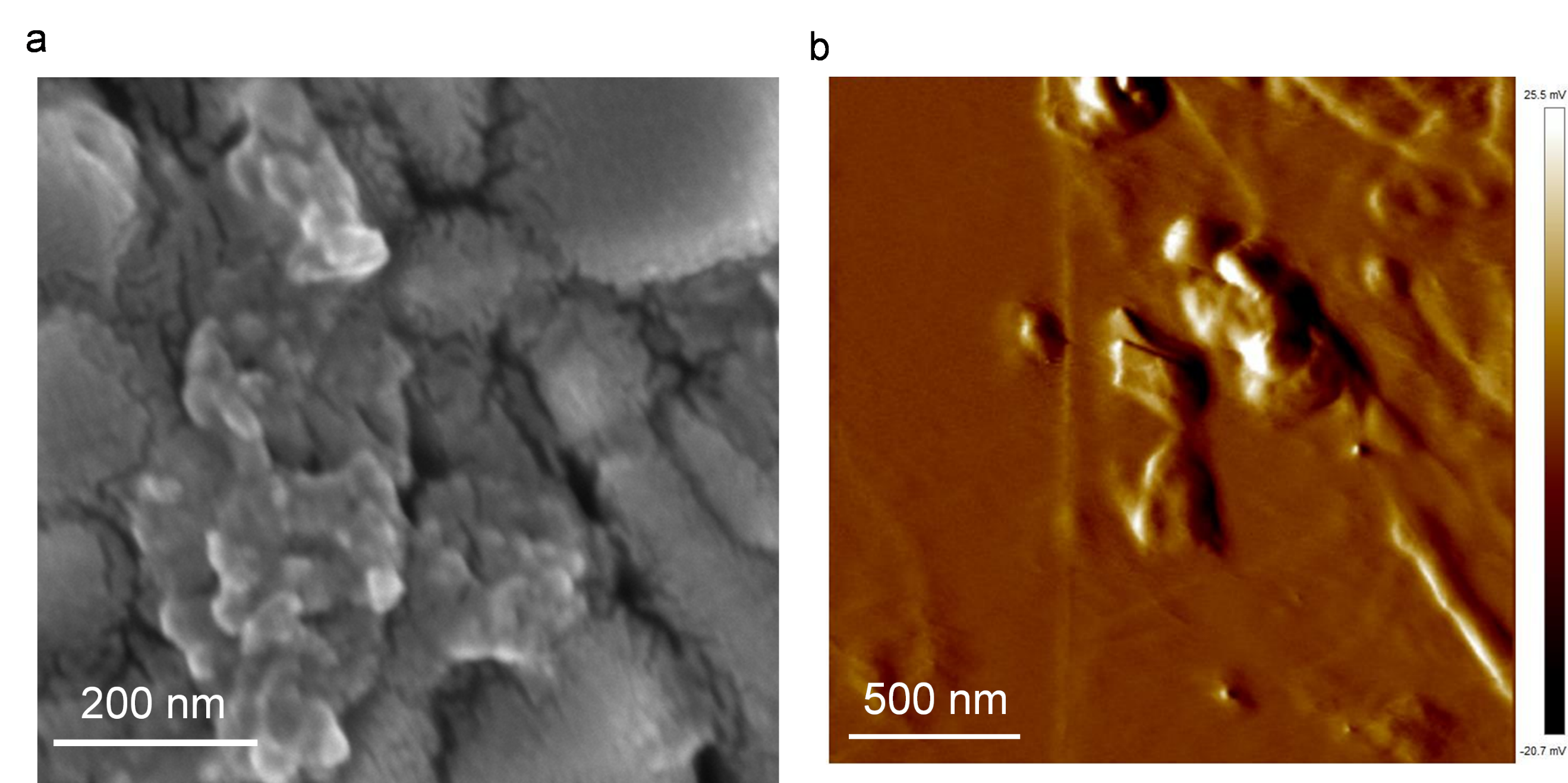


**Supplementary Fig. 10.** SEM and AFM images of CoPc/ppy/GDE.


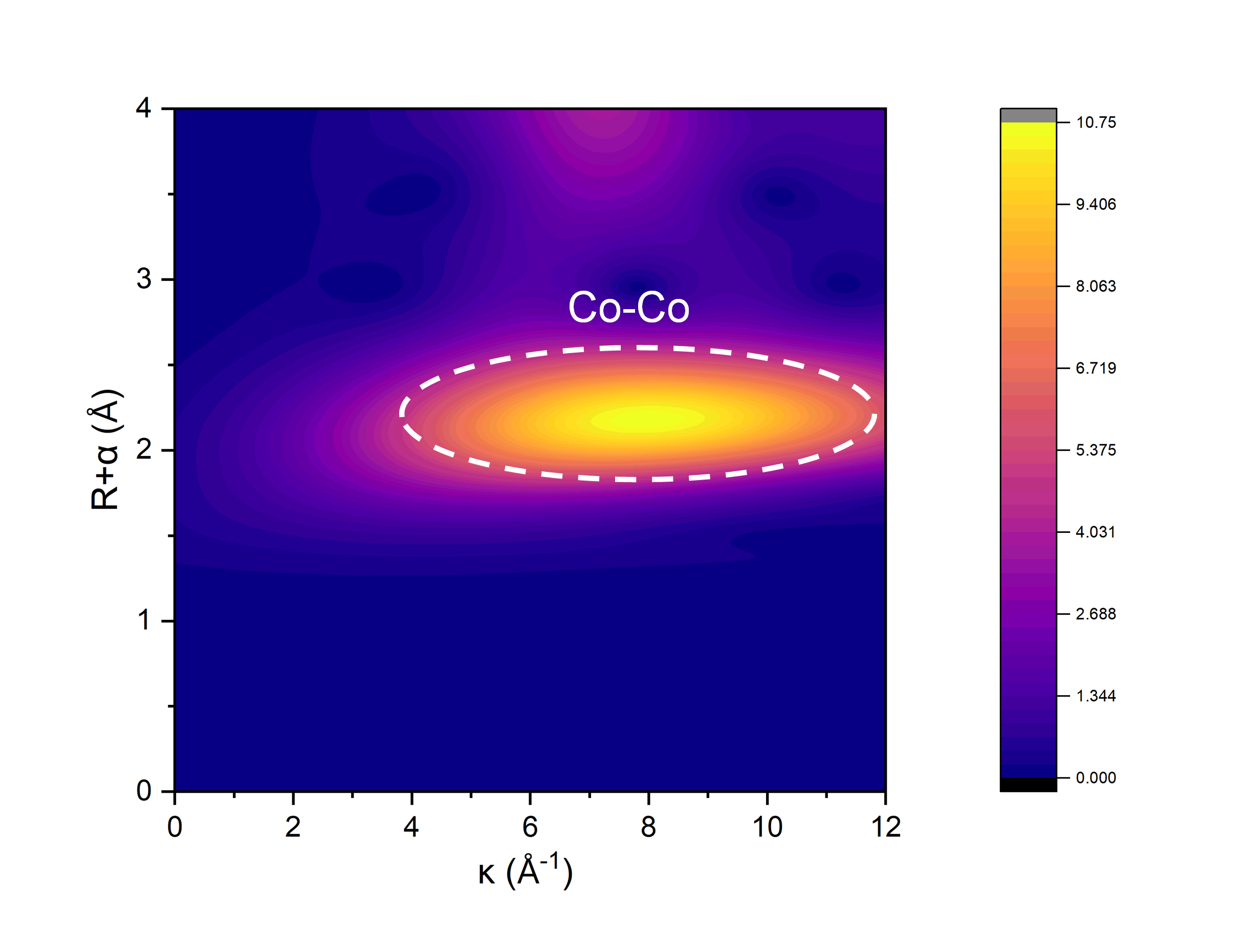


**Supplementary Fig. 11.** WT-EXAFS plots of the Co foil sample.


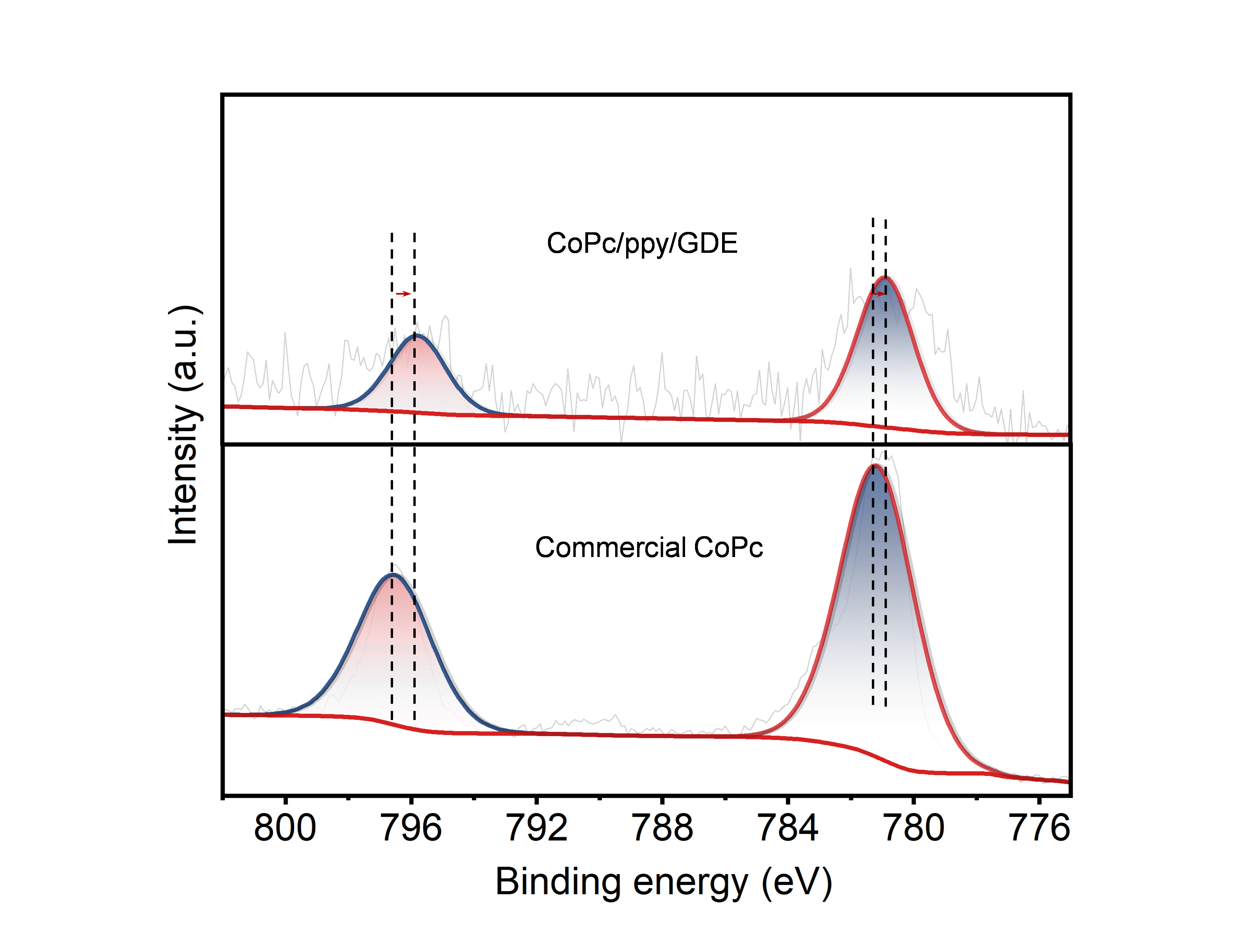


**Supplementary Fig. 12.** Comparison of the Co 2p XPS spectra of CoPc/ppy/GDE and commercial CoPc.


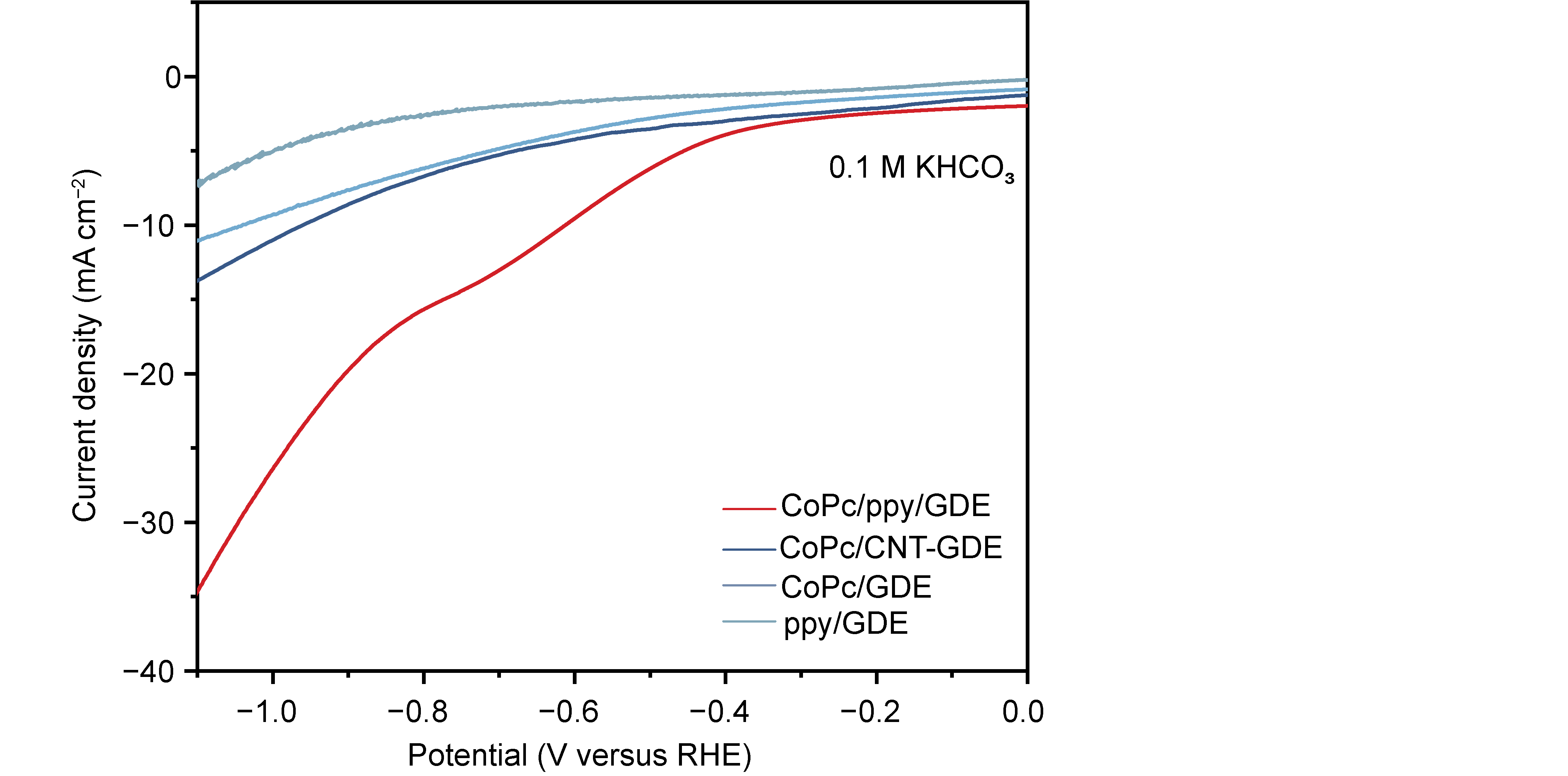


**Supplementary Fig. 13.** LSV curves of CoPc/ppy/GDE, CoPc/CNT-GDE, CoPc/GDE and ppy/GDE during CO_2_RR in CO_2_-saturated 0.1 M KHCO_3_ with an H-type cell. There is no iR correction for voltages. The resistance value of H-cell is 10 ± 2 Ω. The gas flow rate of H-cell is 5 mL/min. All electrochemical measurements were performed at room temperature (23 ± 2℃) in 0.1 M CO_2_-saturated KHCO_3_ solution (pH=6.8 ± 0.2). There is no iR correction for voltages. Source data are provided as a Source Data file.


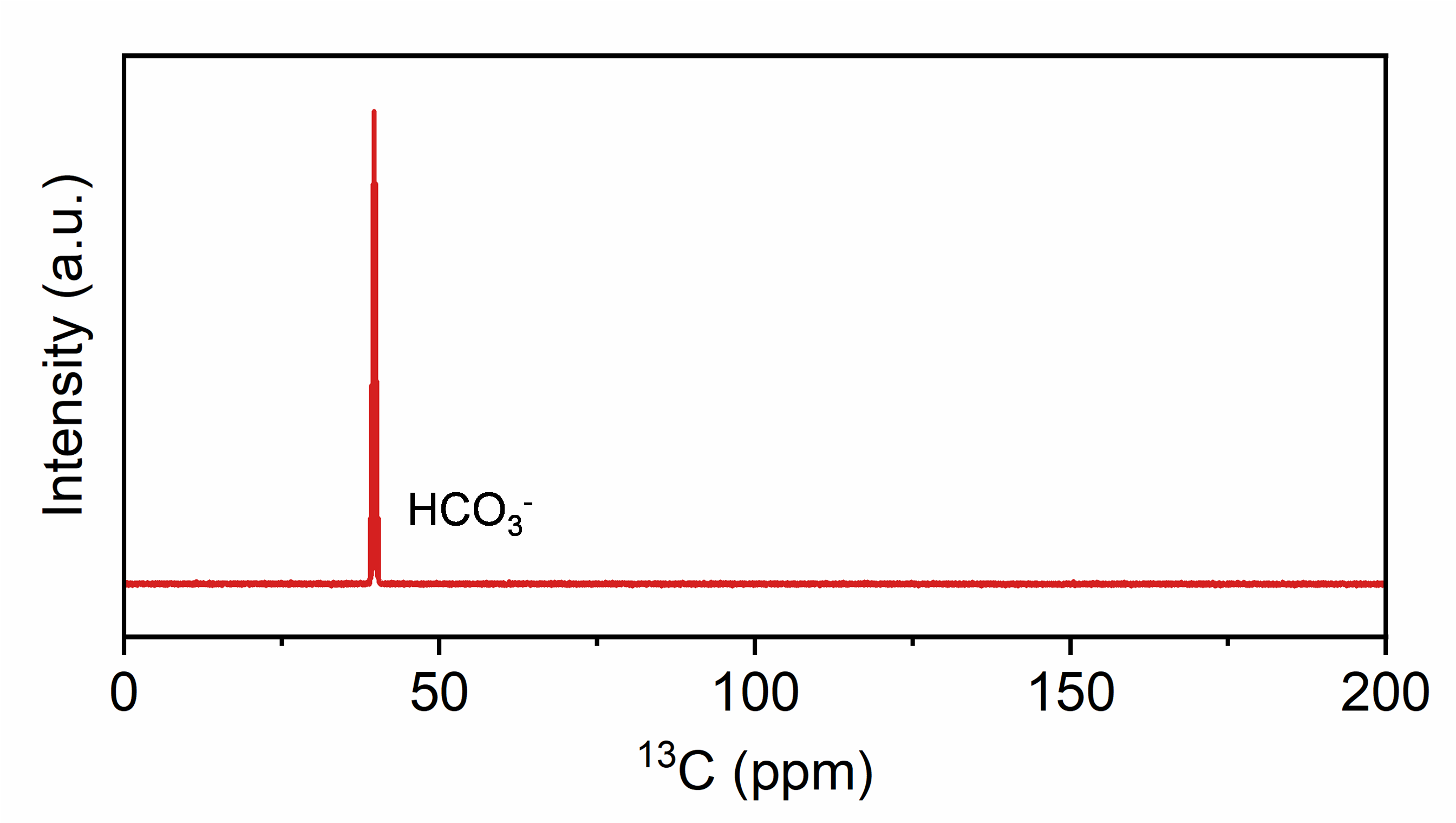


**Supplementary Fig. 14.** ^13^C NMR spectrum of the electrolytes after the 200 hours test.


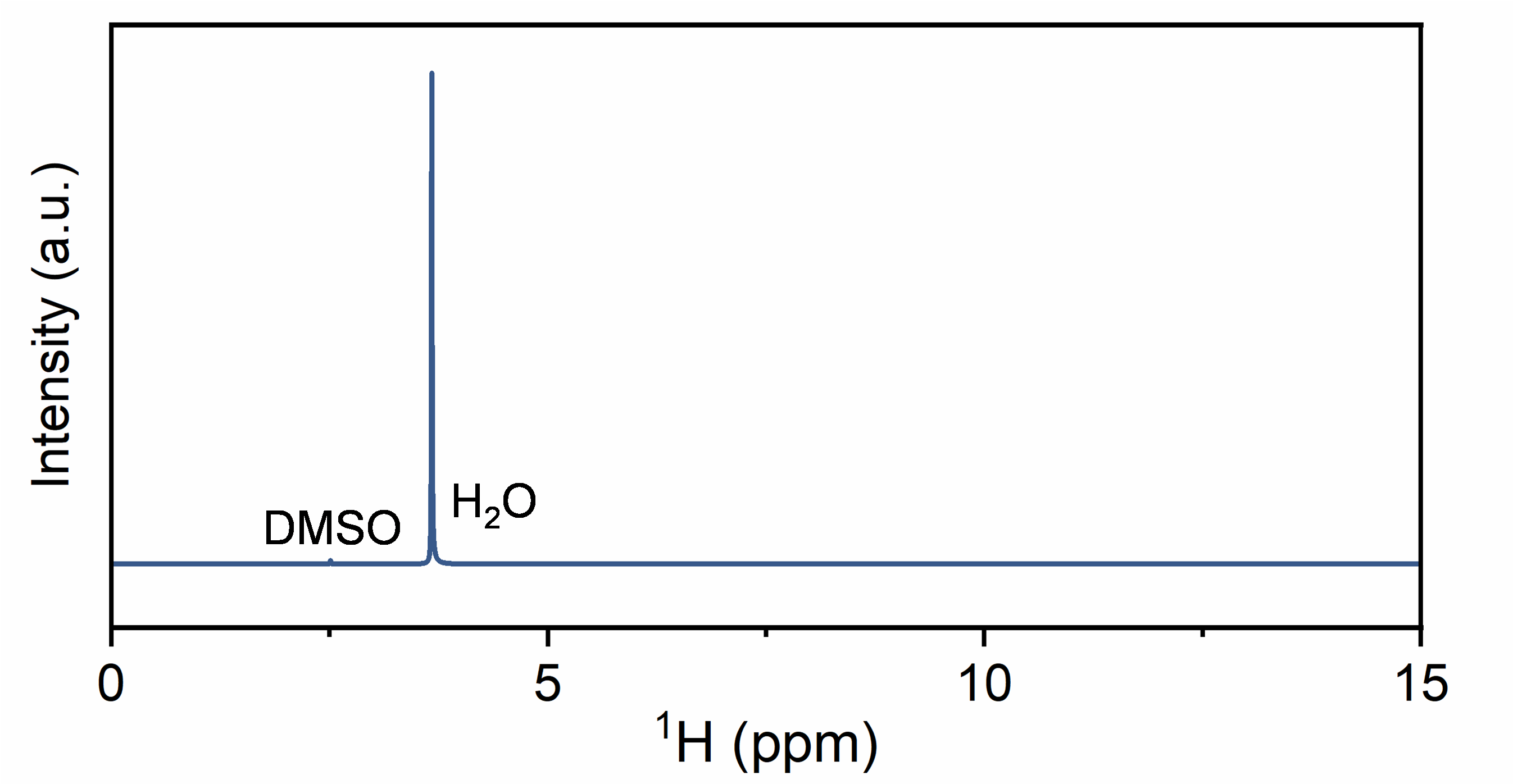


**Supplementary Fig. 15.** ^1^H NMR spectrum of the electrolytes after the 200 hours test.


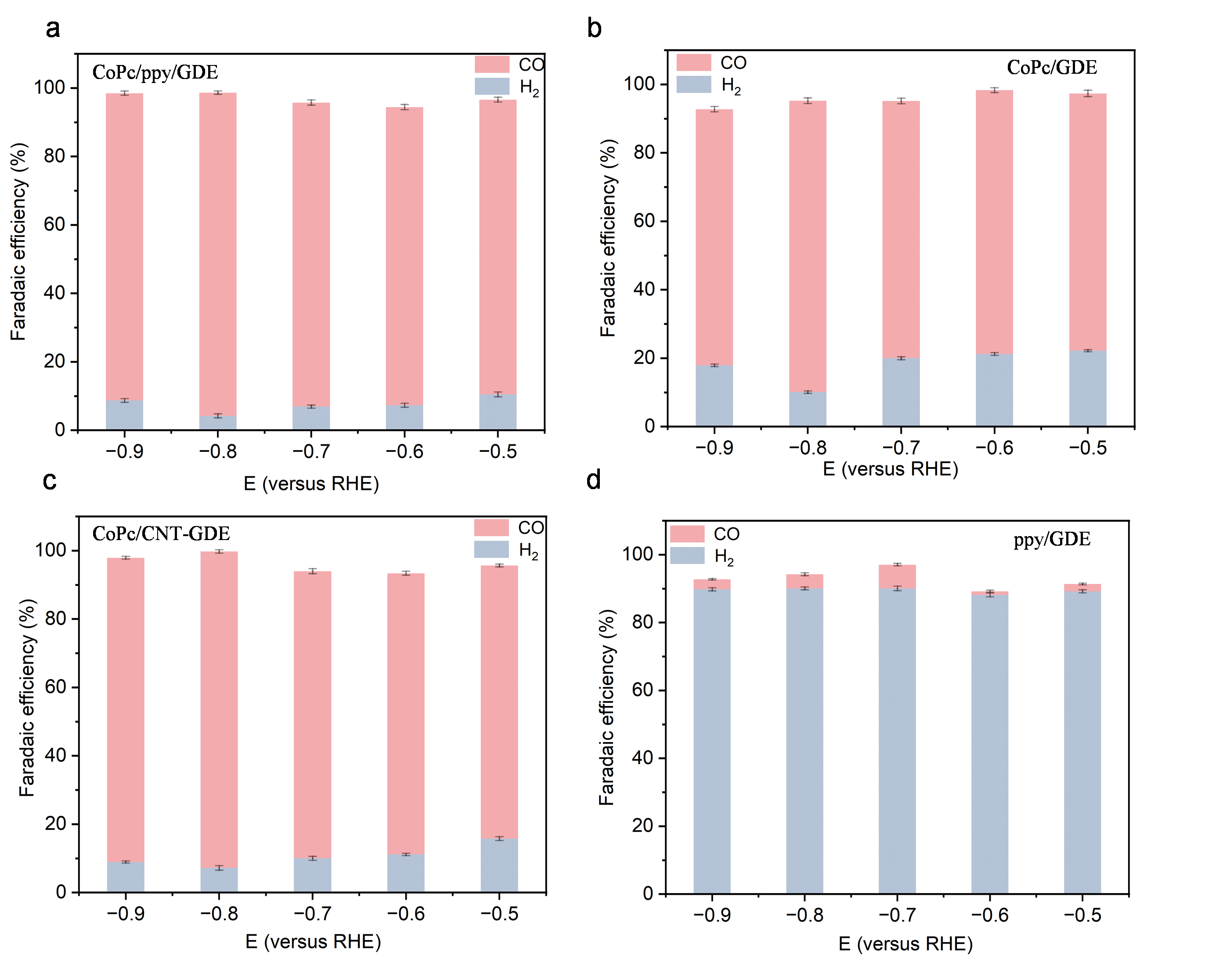


**Supplementary Fig. 16.** Faradaic efficiency of CoPc/ppy/GDE (**a**), CoPc/GDE (**b**), ppy/GDE (**c**) CoPc/CNT-GDE (**d**) during CO_2_RR in CO_2_-saturated 0.1 M KHCO_3_ with an H-type cell. All electrochemical measurements were performed at room temperature (23 ± 2℃). Data are presented as mean values ± standard deviation. The standard deviation is obtained based on three independent samples. Source data are provided as a Source Data file.


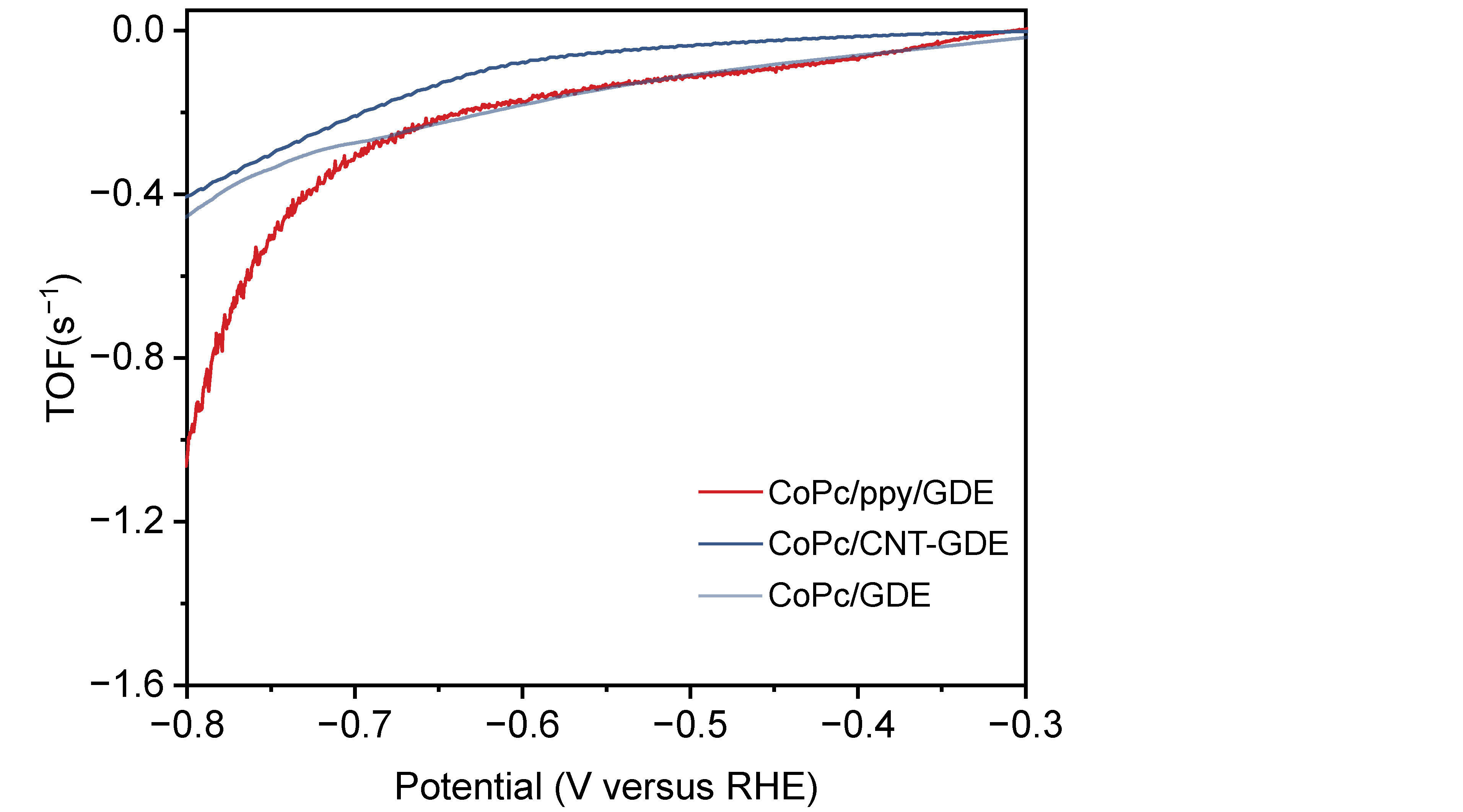


**Supplementary Fig. 17.** TOF_CO_ of CoPc/ppy/GDE, CoPc/CNT-GDE and CoPc/GDE during CO_2_RR in CO_2_-saturated 0.1 M KHCO_3_ with an H-type cell with the scan rate of 1 mV s^−1^. The resistance value of H-cell is 10 ± 2 Ω. The gas flow rate of H-cell is 5 mL/min. All electrochemical measurements were performed at room temperature (23 ± 2℃) in 0.1 M CO_2_-saturated KHCO_3_ solution (pH=6.8 ± 0.2). Source data are provided as a Source Data file.


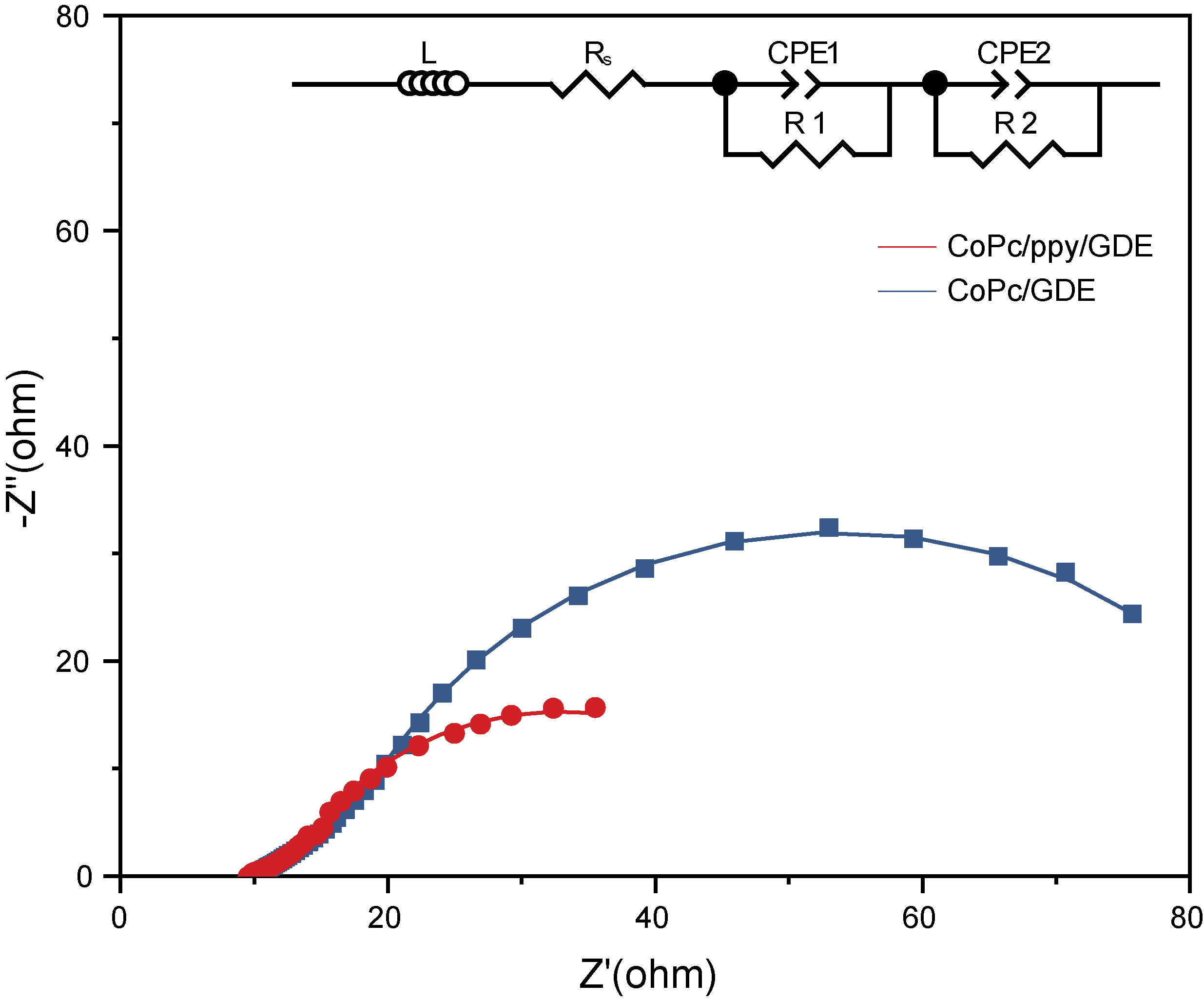


**Supplementary Fig. 18.** The EIS of CoPc/ppy/GDE and CoPc/GDE in CO_2_-saturated 0.1 M KHCO_3_ with an H-type cell at −0.1 V versus RHE. The resistance value of H-cell is 10 ± 2 Ω. The gas flow rate of H-cell is 5 mL/min. All electrochemical measurements were performed at room temperature (23 ± 2℃) . There is no iR correction for voltages. Source data are provided as a Source Data file.

To better understand the associated electrochemical processes occurring in CoPc/ppy/GDE, EIS was measured at −0.1 V vs. RHE. As shown in supplementary Fig. 17, CoPc/ppy/GDE and CoPc/GDE exhibit comparable solution resistances R1 of 10.59 Ω and 9.81 Ω, respectively. Equivalent circuit modeling yields a much lower electrode resistance R2 of 4.53 Ω for CoPc/ppy/GDE than that of CoPc/GDE (9.68 Ω), as well as an almost halved interfacial charge transfer resistance R3 (38.47 Ω versus 69.20 Ω; Supplementary Table 3).


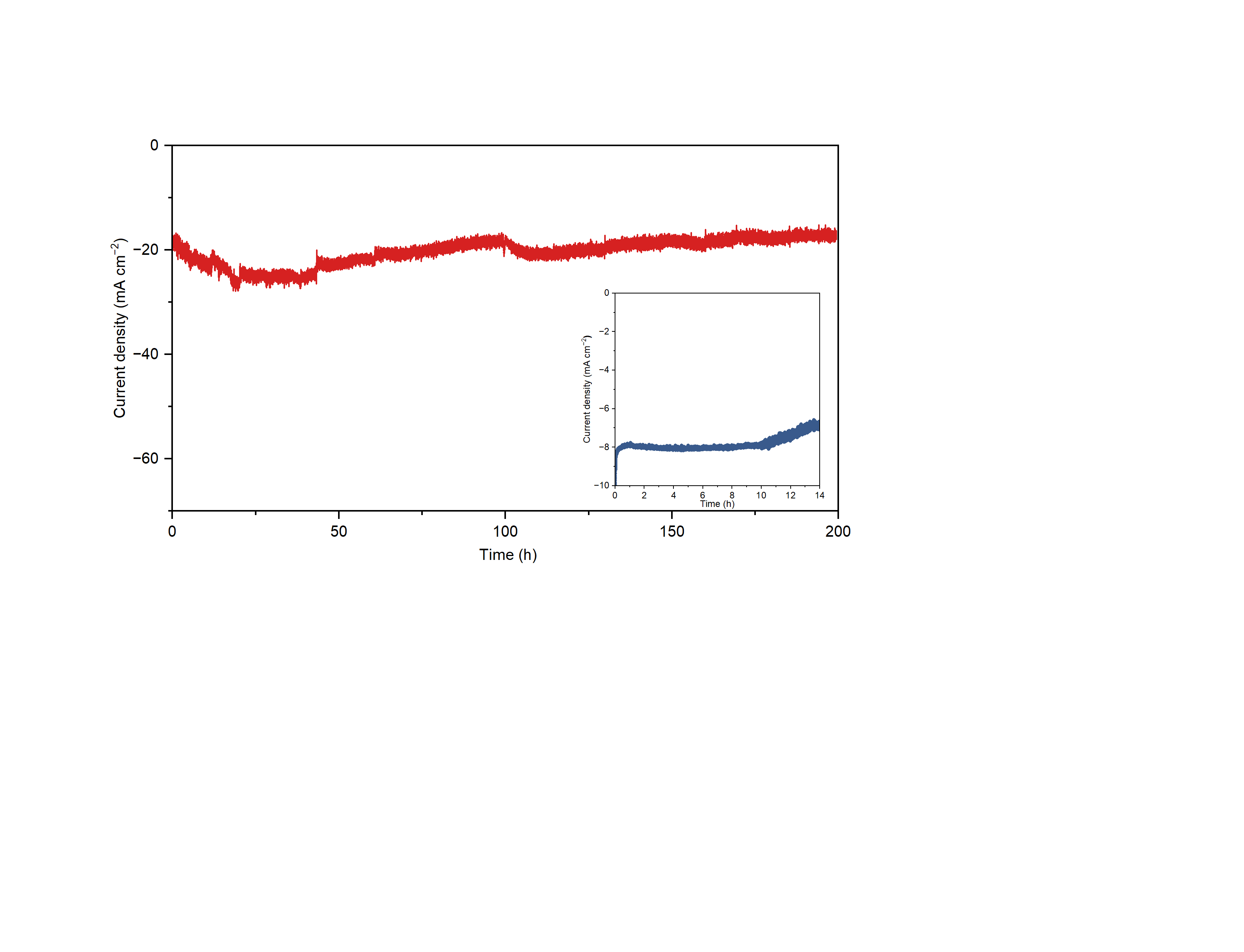


**Supplementary Fig. 19.** Long-term stability of the CoPc/ppy/GDE and CoPc/CNT-GDE (inset) in CO_2_-saturated 0.1 M KHCO_3_ with an H-type cell at −0.75 V versus RHE. The resistance value of H-cell is 10 ± 2 Ω. The gas flow rate of H-cell is 5 mL/min. All electrochemical measurements were performed at room temperature (23 ± 2℃). Source data are provided as a Source Data file.


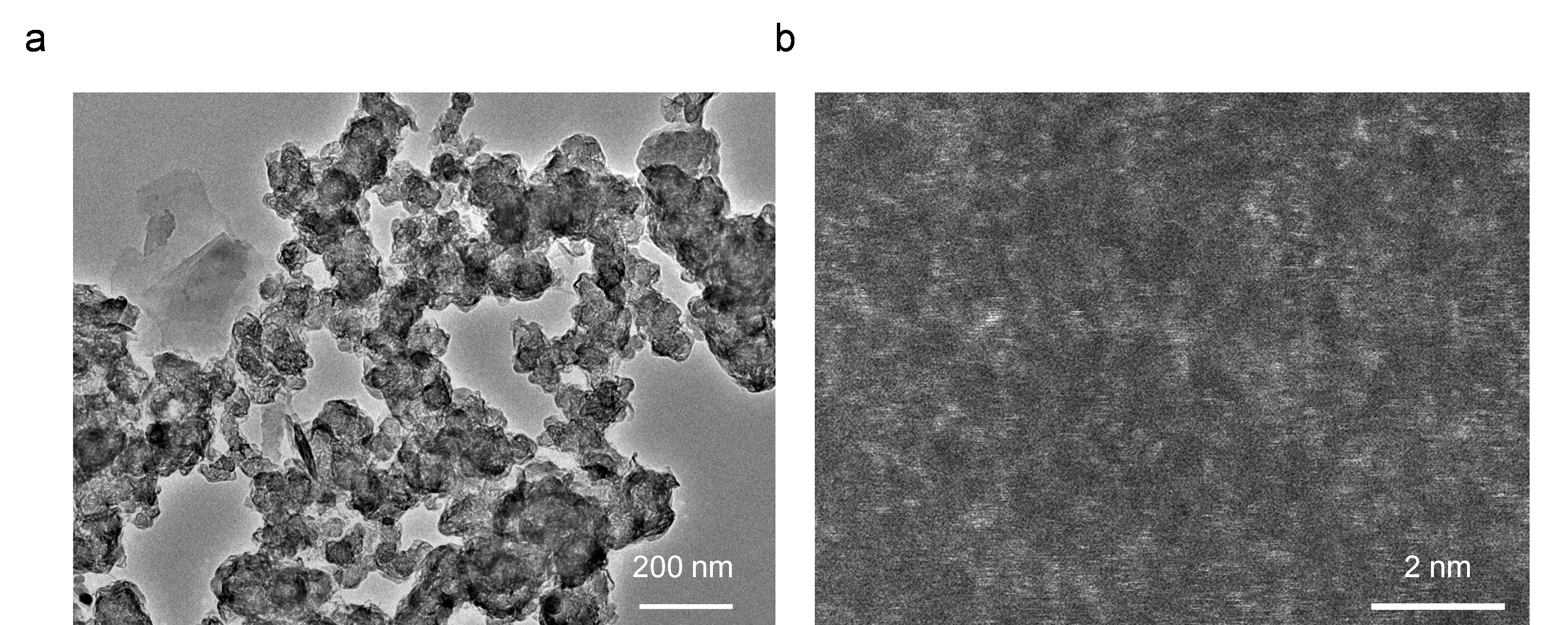


**Supplementary Fig. 20.** **a**, TEM image of post-reacted CoPc/ppy/GDE after 200 hours. **b**, HAADF-STEM image of post-reacted CoPc/ppy/GDE after 200 hours.


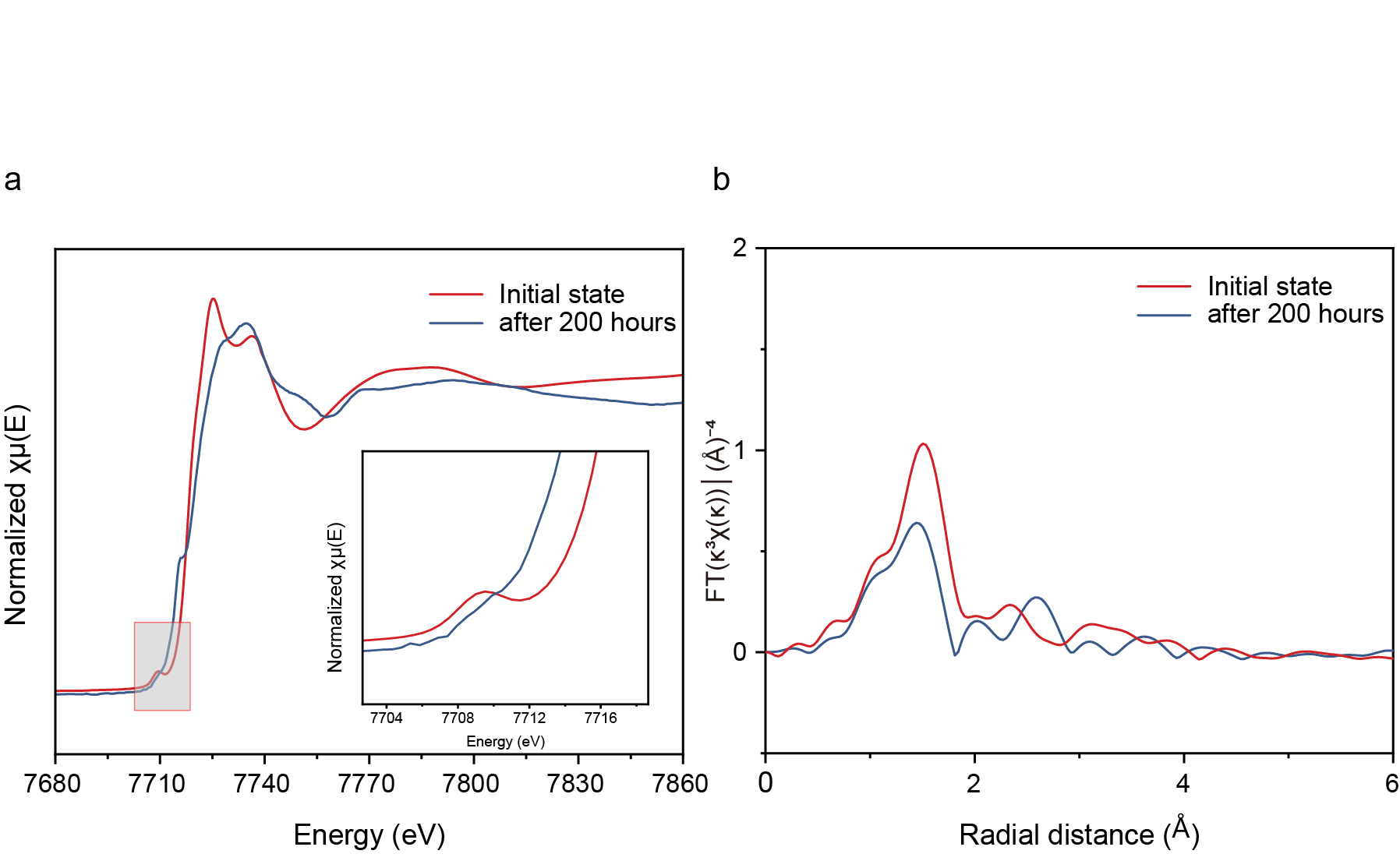


**Supplementary Fig. 21.** **a**, XAS spectra at Co K-edge of CoPc/ppy/GDE and **b**, Co K-edge FT EXAFS spectra in the R space of CoPc/ppy/GDE of initial state and after 200 hours in CO_2_-saturated 0.1 M KHCO_3_ with an H-type cell at −0.75 V versus RHE.


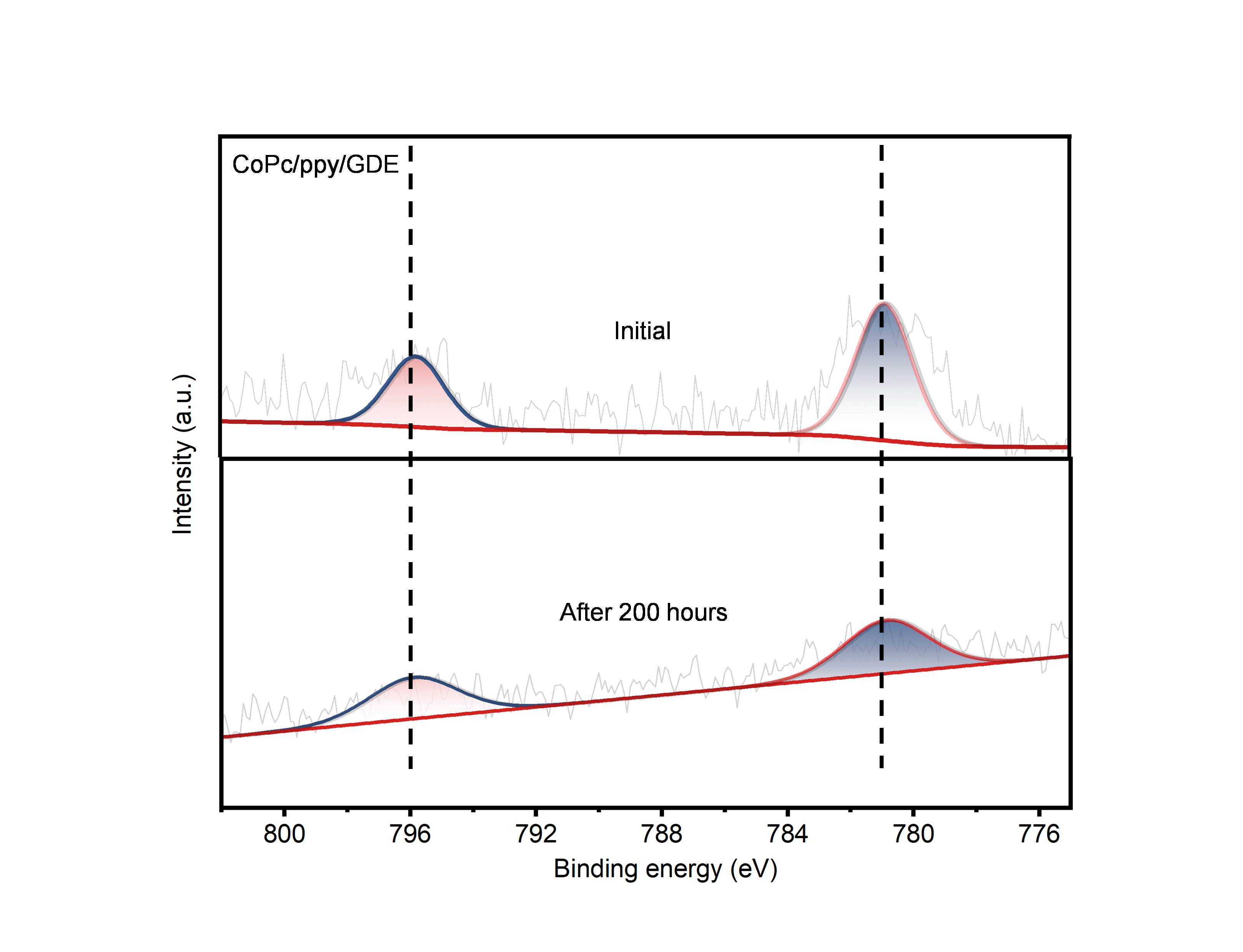


**Supplementary Fig. 22.** Comparison of the Co 2p XPS spectra of CoPc/ppy/GDE of initial state and after 200 hours in CO_2_-saturated 0.1 M KHCO_3_ with an H-type cell at −0.75 V versus RHE.


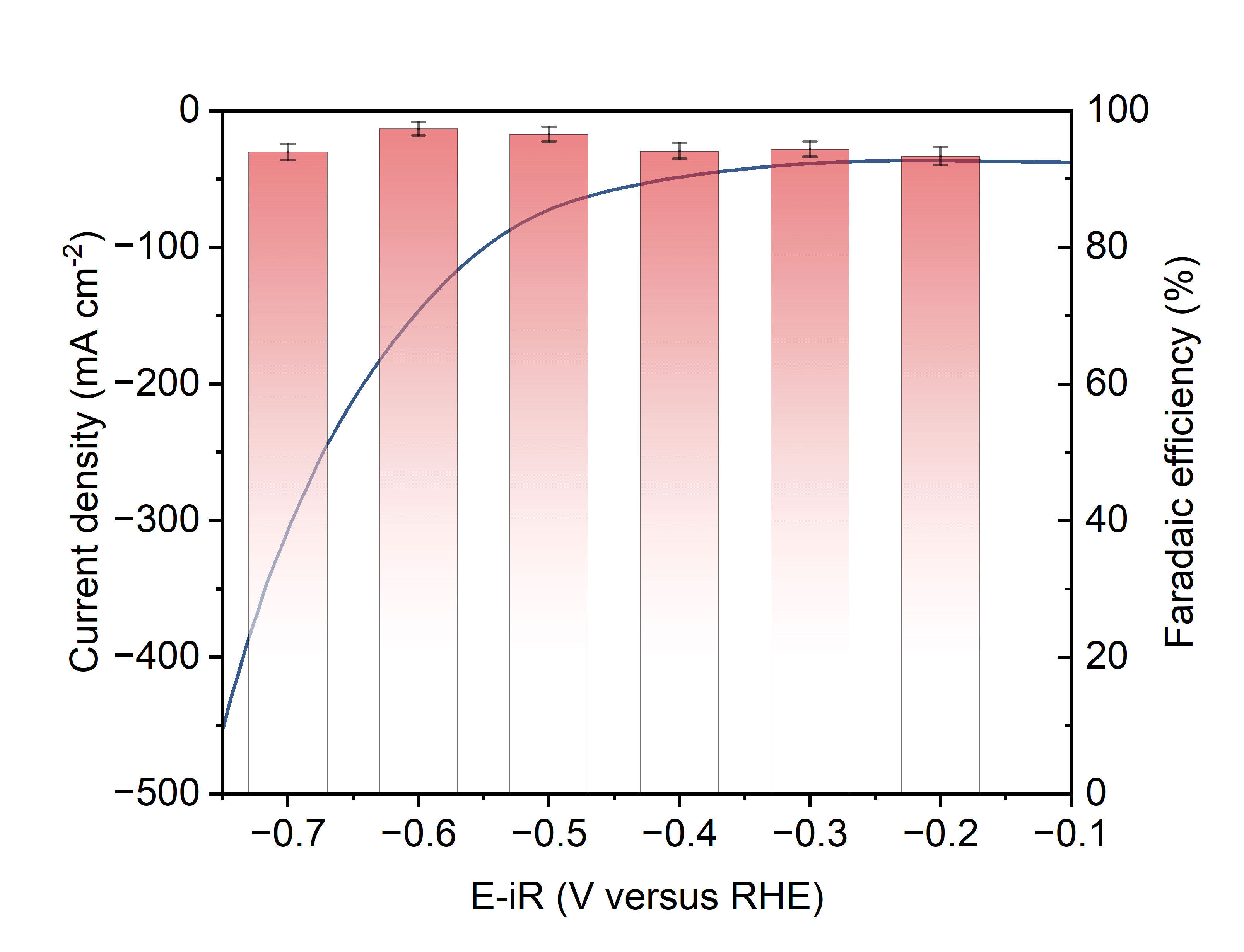


**Supplementary Fig. 23.** LSV and Faradaic efficiency of CoPc/ppy/GDE during CO_2_RR in a flow cell with 1.0 M KOH electrolyte. The resistance value of flow cell is 1 ± 0.4 Ω. The gas flow rate of H-cell is 20 mL/min. All electrochemical measurements were performed at room temperature (23 ± 2℃). Data are presented as mean values ± standard deviation. The standard deviation is obtained based on three independent samples. Source data are provided as a Source Data file.


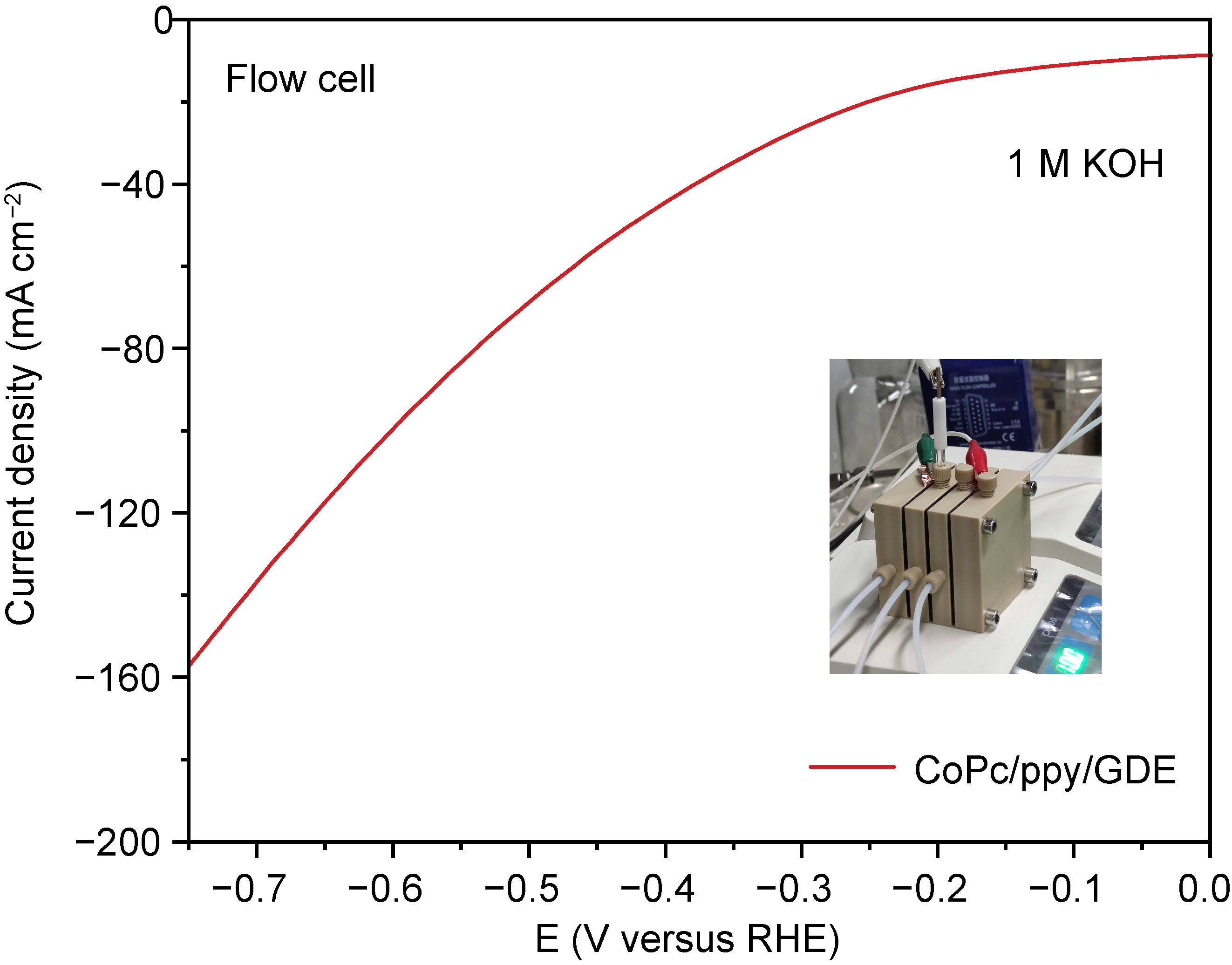


**Supplementary Fig. 24.** LSV of CoPc/ppy/GDE during CO_2_RR in a flow cell with 1.0 M KOH electrolyte. The resistance value of flow cell is 1 ± 0.4 Ω. The gas flow rate of H-cell is 20 mL/min. All electrochemical measurements were performed at room temperature (23 ± 2℃). There is no iR correction for voltages. Source data are provided as a Source Data file.


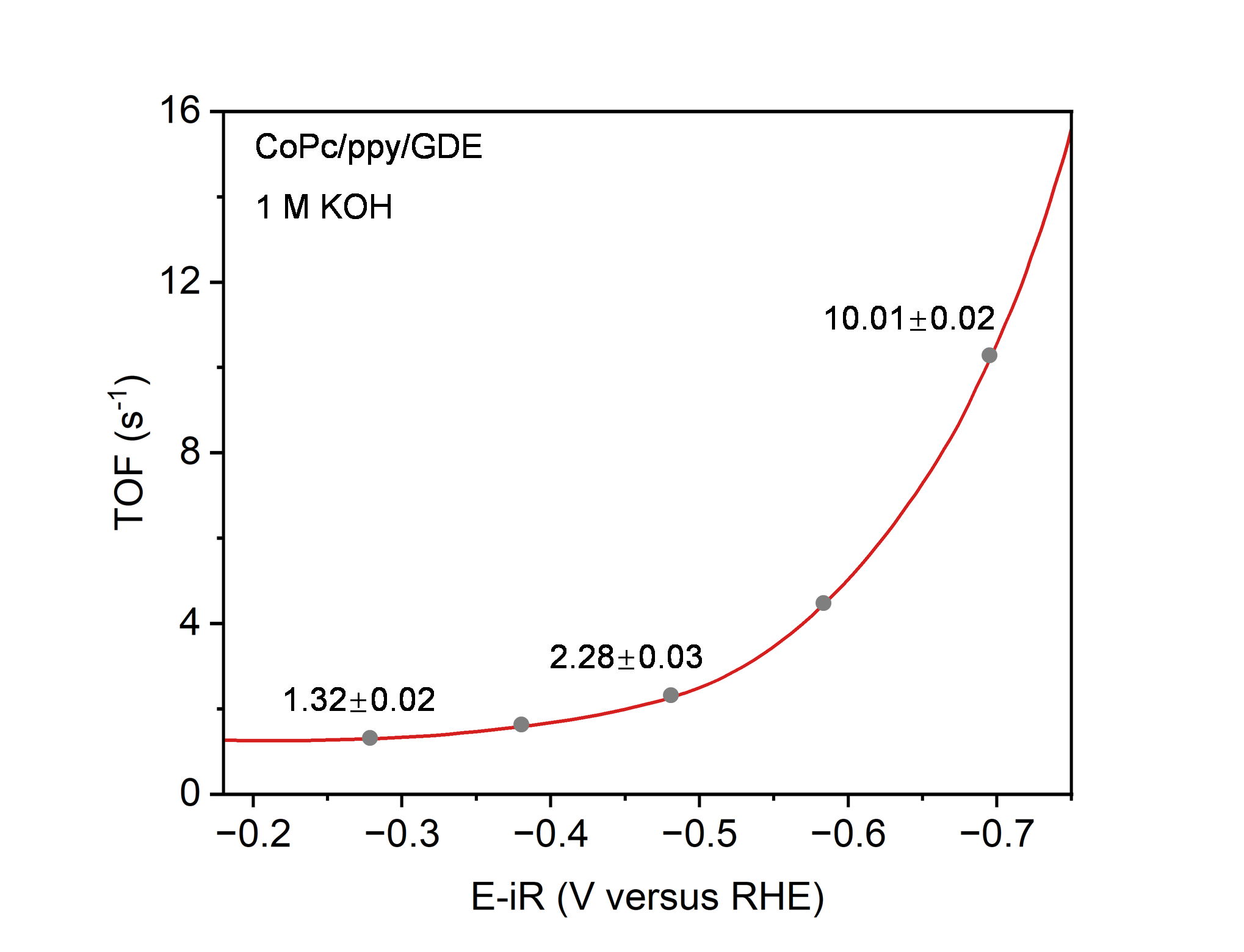


**Supplementary Fig. 25.** TOF_CO_ of CoPc/ppy/GDE during CO_2_RR in a flow cell with 1.0 M KOH electrolyte. The resistance value of flow cell is 1 ± 0.4 Ω. The gas flow rate of H-cell is 20 mL/min. All electrochemical measurements were performed at room temperature (23 ± 2℃). Source data are provided as a Source Data file.


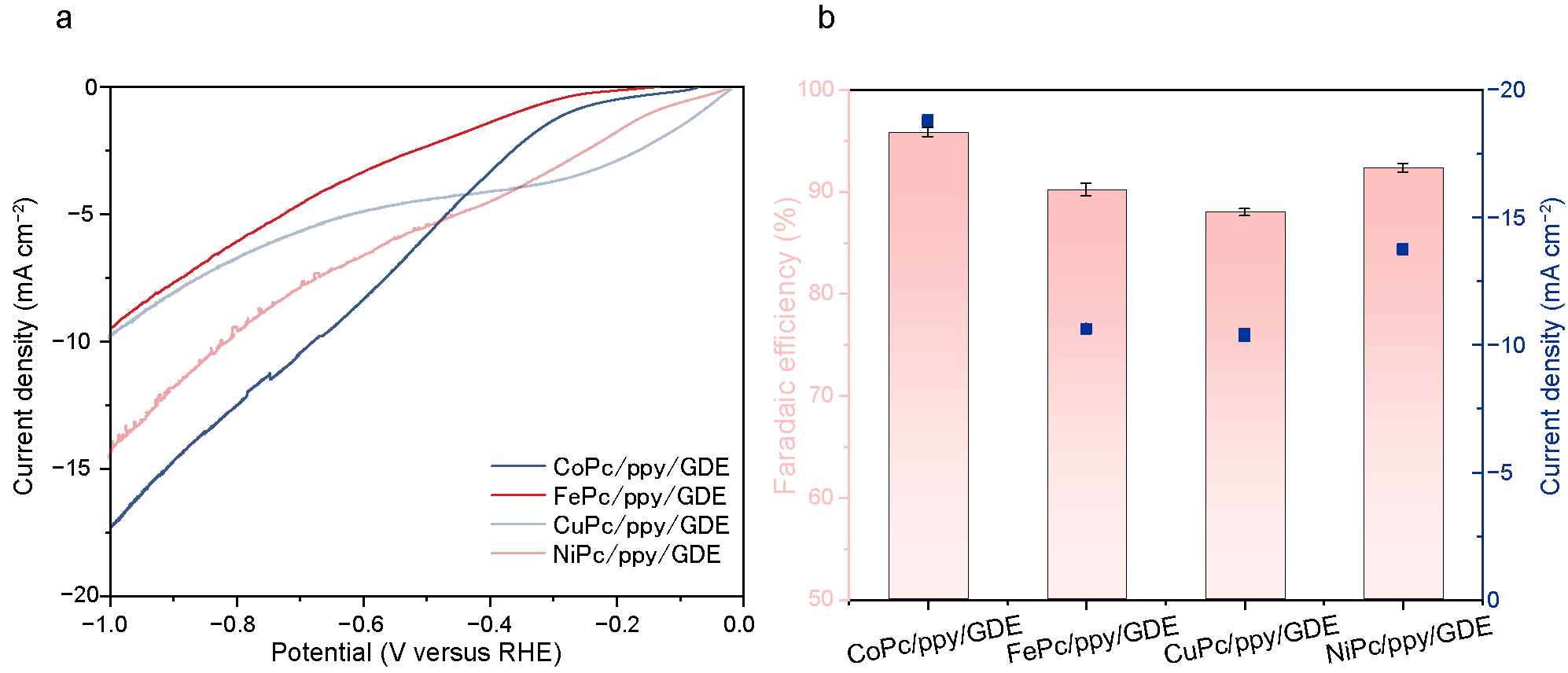


**Supplementary Fig. 26.** **a**, LSV curves of CoPc/ppy/GDE, FePc/ppy/GDE, CuPc/ppy/GDE and NiPc/ppy/GDE during CO_2_RR in CO_2_-saturated 0.1 M KHCO_3_ with an H-type cell. **b**, Faradaic efficiency and current density of CoPc/ppy/GDE, FePc/ppy/GDE, CuPc/ppy/GDE and NiPc/ppy/GDE during CO_2_RR in CO_2_-saturated 0.1 M KHCO_3_ with an H-type cell at −1.0 V versus RHE. The resistance value of H-cell is 10 ± 2 Ω. The gas flow rate of H-cell is 5 mL/min. All electrochemical measurements were performed at room temperature (23 ± 2℃). There is no iR correction for voltages. Data are presented as mean values ± standard deviation. The standard deviation is obtained based on three independent samples. Source data are provided as a Source Data file.


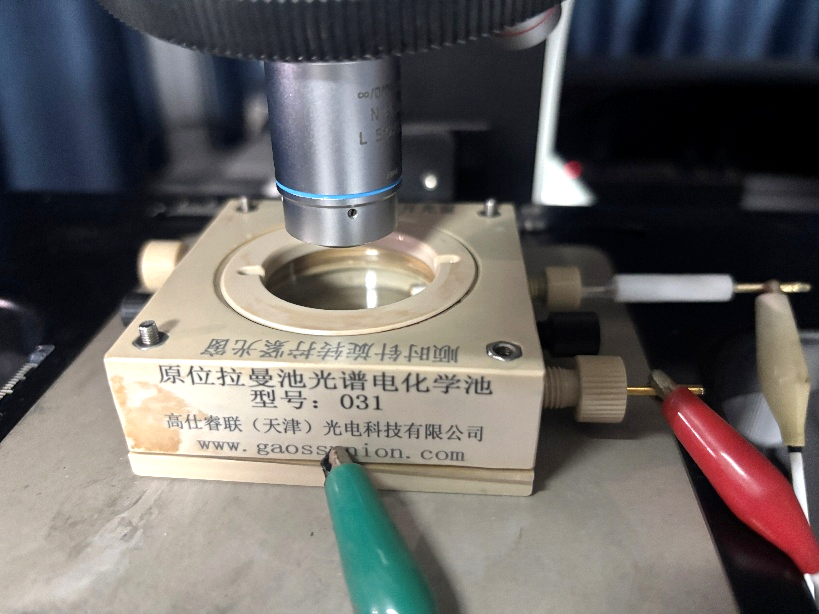


**Supplementary Fig. 27.** The photograph of the electrochemical cell for in situ Raman measurements.


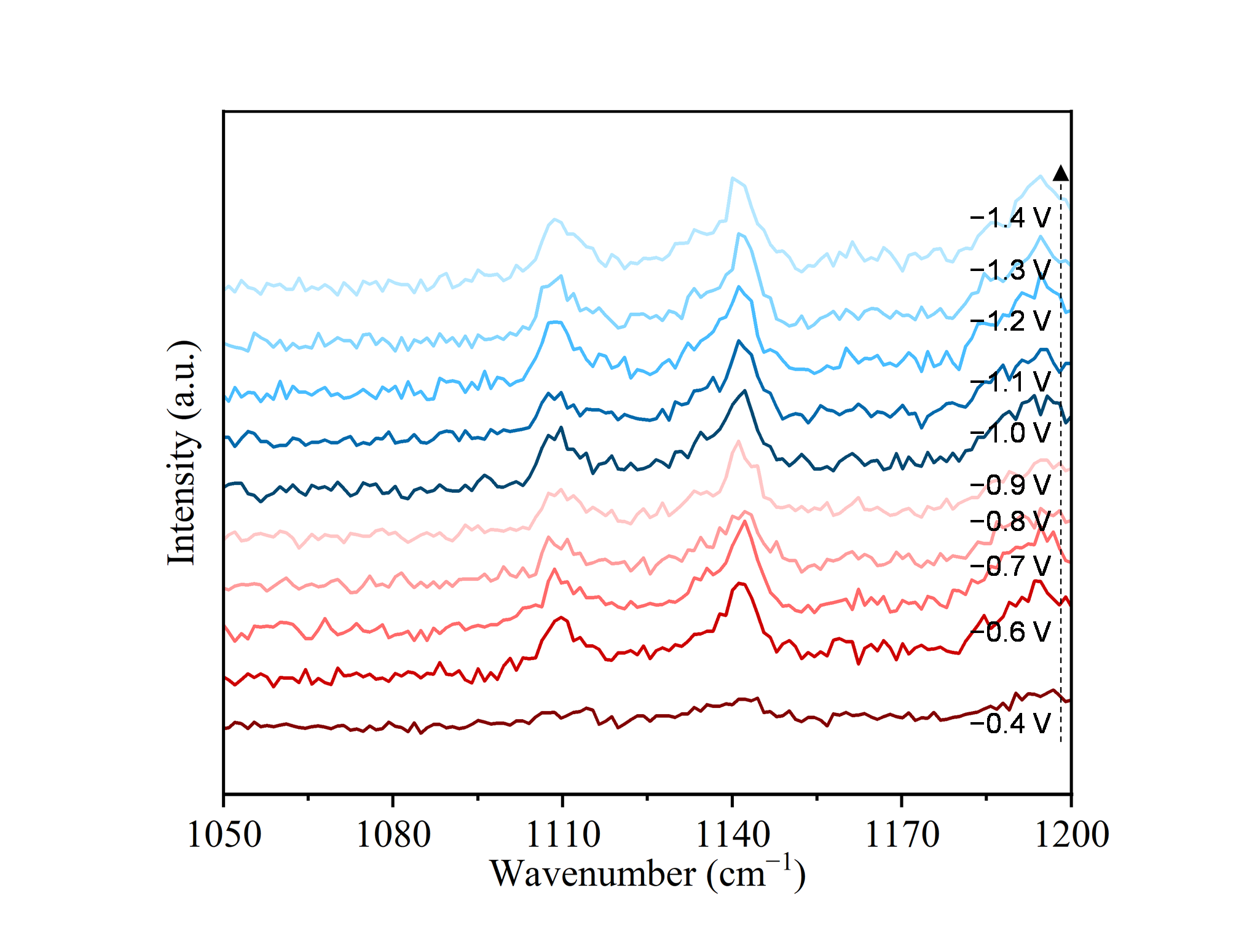


**Supplementary Fig. 28.** In situ surface-enhanced Raman spectra of the CoPc/ppy/GDE during CO_2_RR under applied bias ranging from the −0.4 to −1.4 V versus RHE in CO_2_-saturated 0.1 M KHCO_3_.


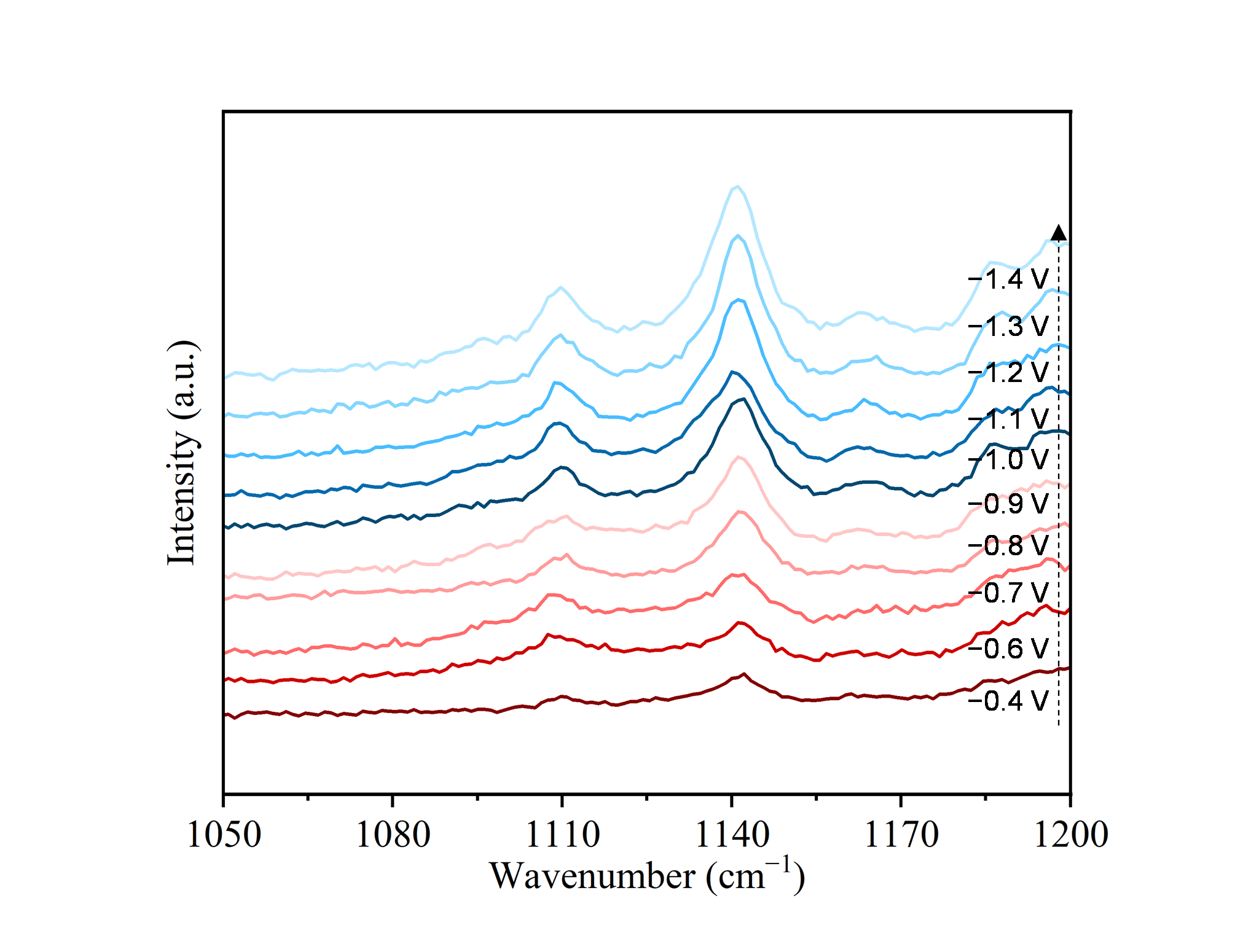


**Supplementary Fig. 29.** In situ surface-enhanced Raman spectra of the CoPc/CNT-GDE during CO_2_RR under applied bias ranging from the −0.4 to −1.4 V versus RHE in CO_2_-saturated 0.1 M KHCO_3_.

.


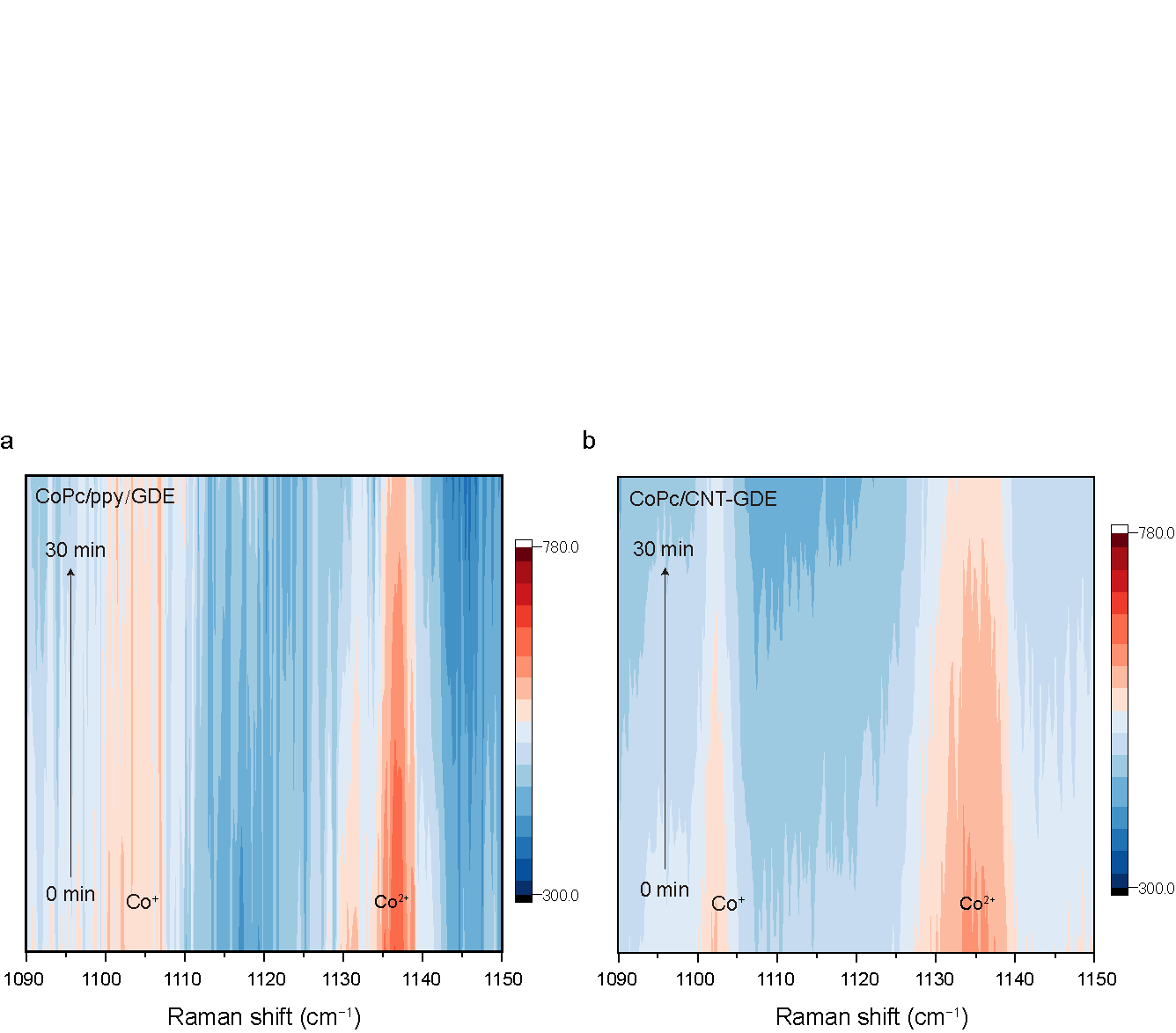


**Supplementary Fig. 30.** Time-dependent in situ surface-enhanced Raman spectra of the **a** CoPc/ppy/GDE, **b** CoPc/CNT-GDE during CO_2_RR from 0 to 30 min at −1.4 V versus RHE in CO_2_-saturated 0.1 M KHCO_3_.


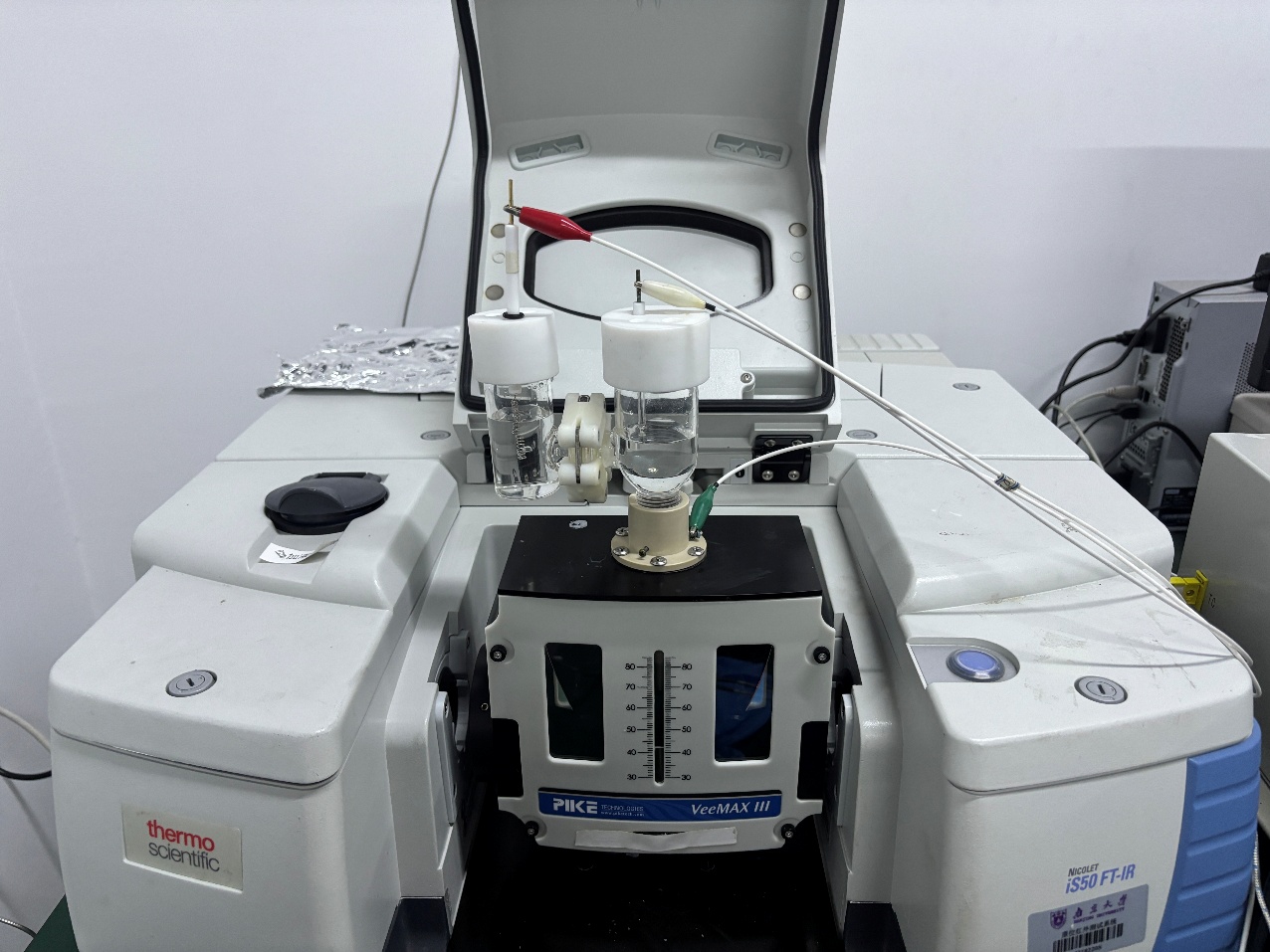


**Supplementary Fig. 31.** The photograph of the electrochemical cell for in situ ATR-SEIRAS measurements.


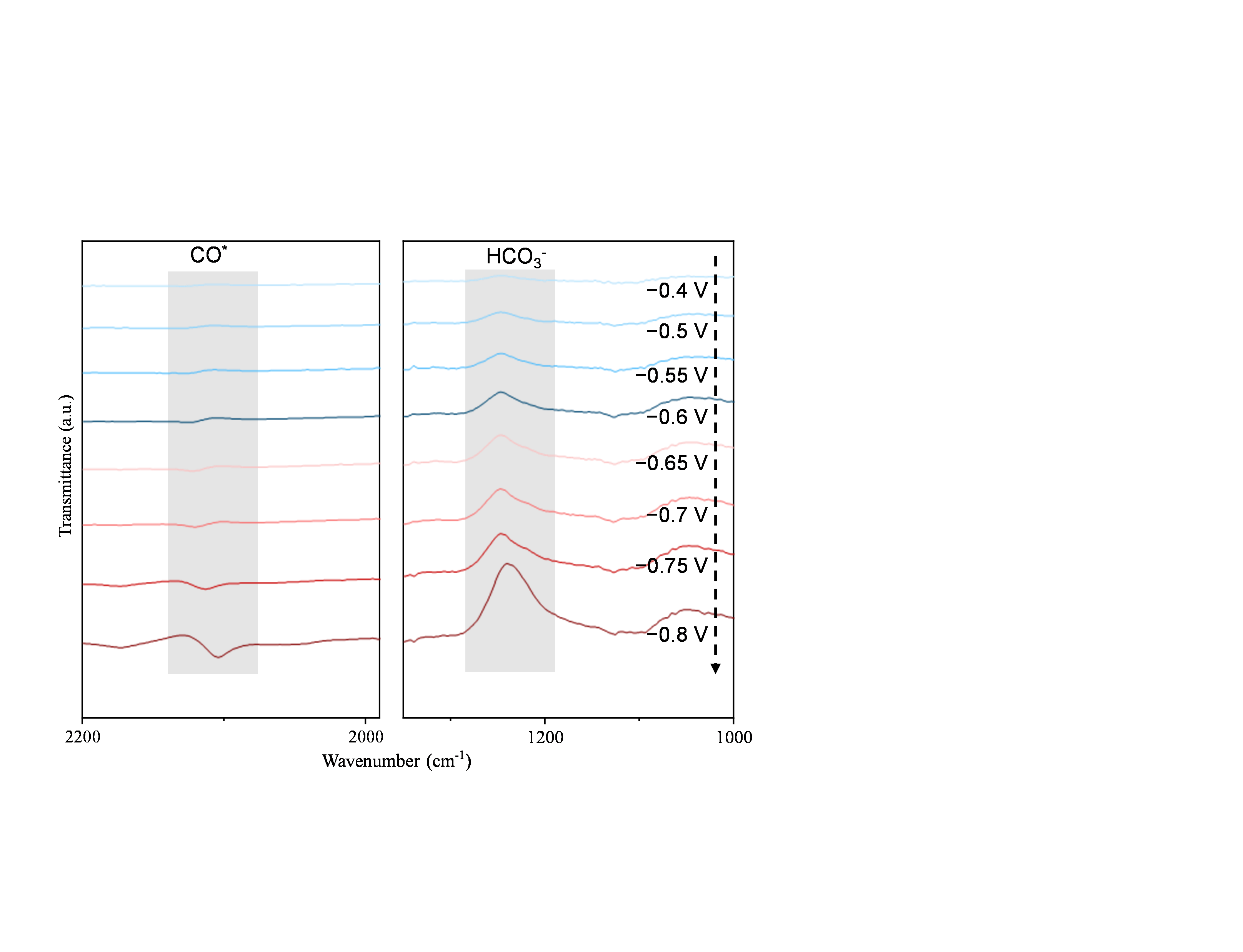


**Supplementary Fig. 32.** In situ ATR-SEIRAS spectra of CoPc/CNT-GDE collected in CO_2_-saturated 0.1 M KHCO_3_ with an H-type cell, the potential was applied from −0.4 to −0.8 V versus RHE.


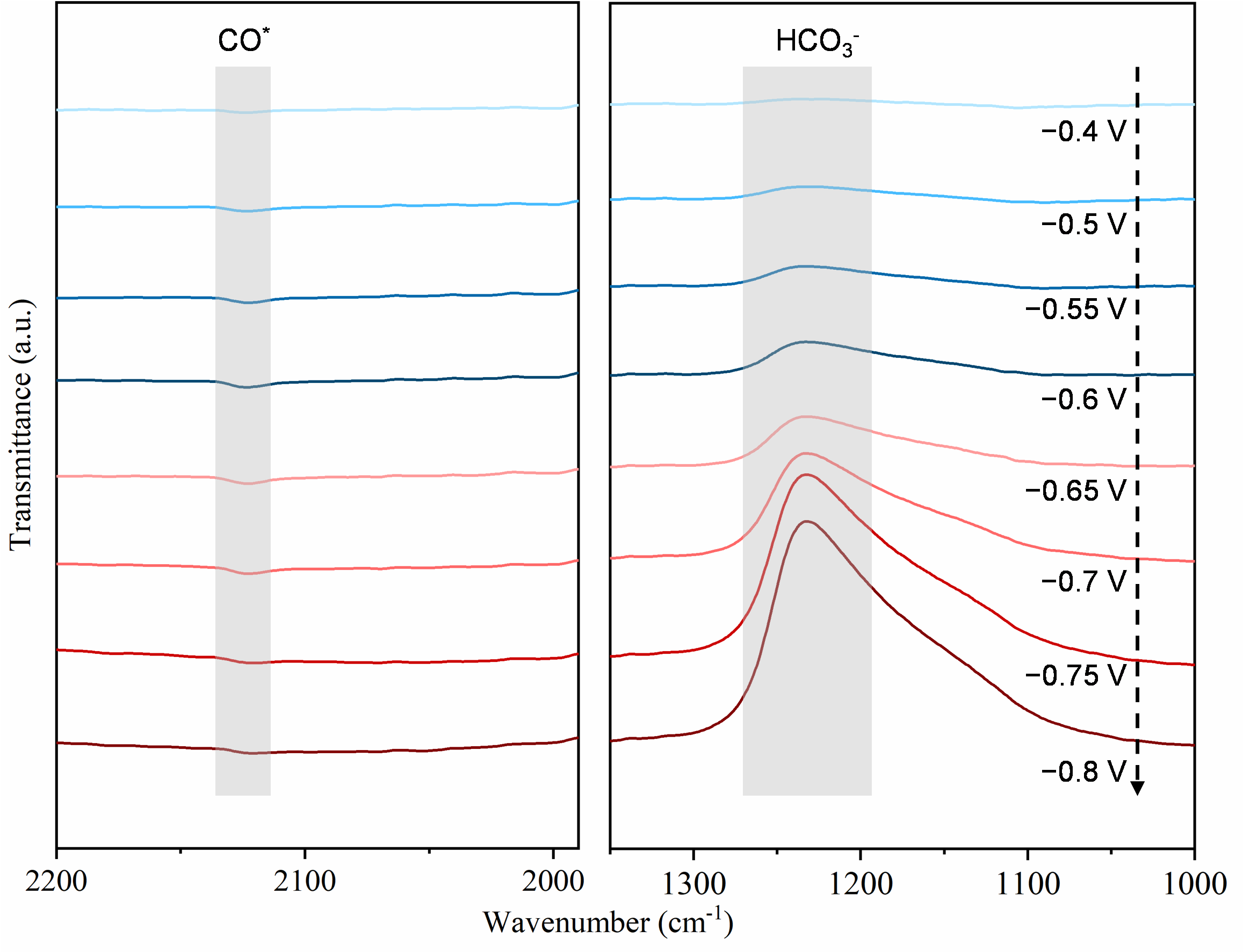


**Supplementary Fig. 33.** In situ ATR-SEIRAS spectra of CoPc/GDE collected in CO_2_-saturated 0.1 M KHCO_3_ with an H-type cell, the potential was applied from −0.4 to −0.8 V versus RHE.


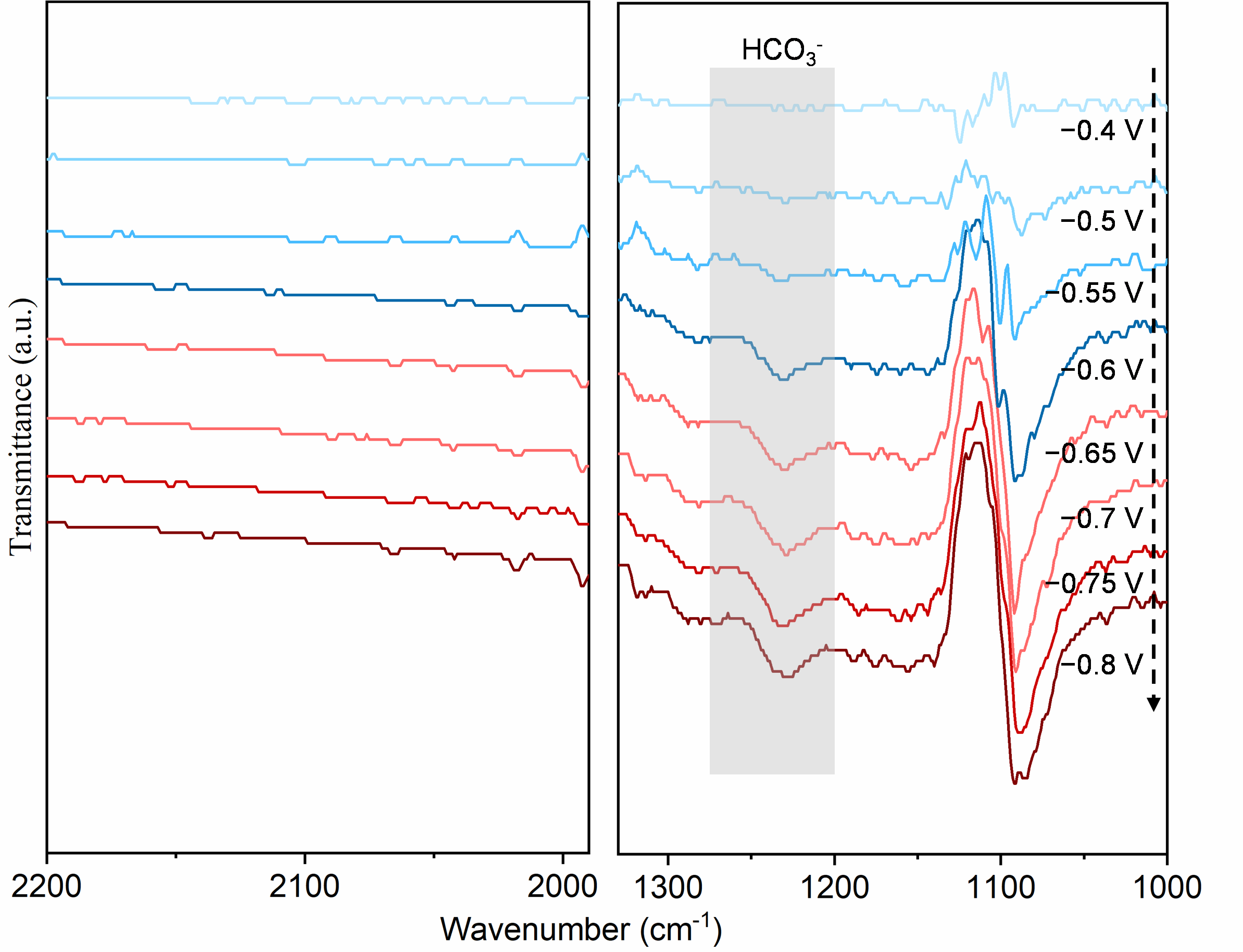


**Supplementary Fig. 34.** In situ ATR-SEIRAS spectra of ppy/GDE collected in CO_2_-saturated 0.1 M KHCO_3_ with an H-type cell, the potential was applied from −0.4 to −0.8 V versus RHE.


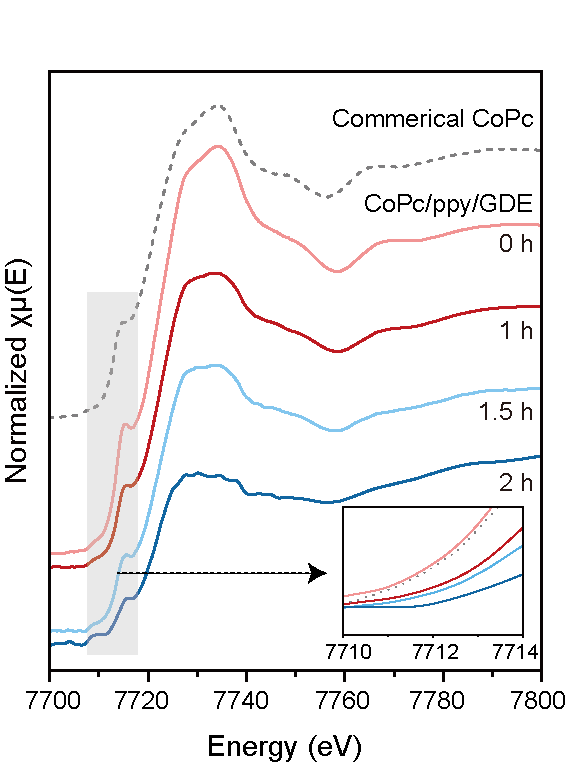


**Supplementary Fig. 35.** Co K-edge XANES spectra of CoPc/ppy/GDE polarized at −0.8 V versus RHE for different reaction periods; commercial CoPc was used as the reference. Note that these spectra were collected under ex situ conditions, during the CO_2_-to-CO conversion the CoPc/ppy/GDE electrode was taken out from the electrochemical cell at designated times and subjected to measurements.


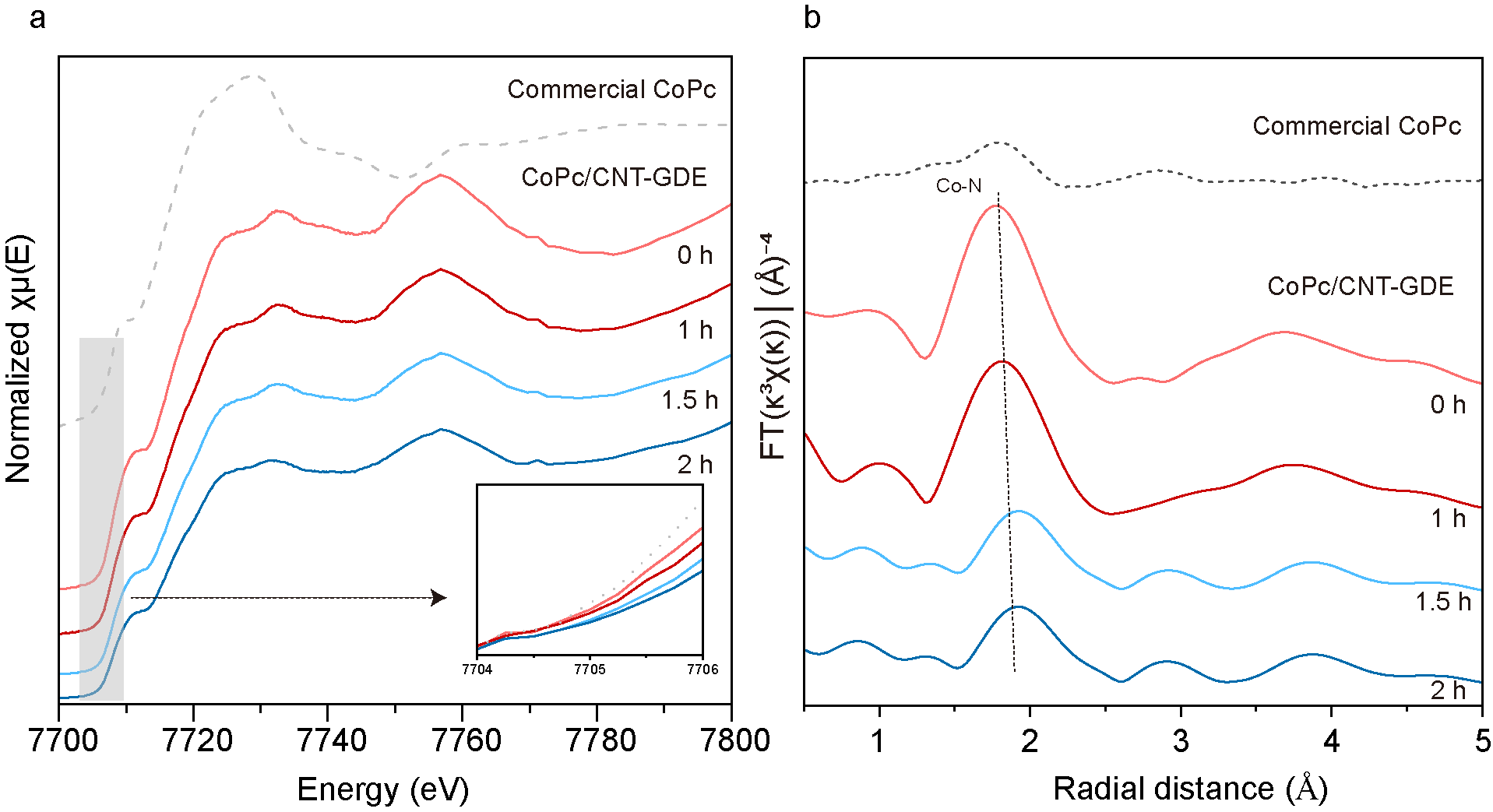


**Supplementary Fig. 36.** Co K-edge (**a**) XANES and (**b**) EXAFS spectra of CoPc/CNT-GDE polarized at −0.8 V versus RHE for different reaction periods; commercial CoPc was used as the reference. Note that these spectra were collected under ex situ conditions, during the CO_2_-to-CO conversion the CoPc/CNT-GDE electrode was taken out from the electrochemical cell at designated times and subjected to measurements.


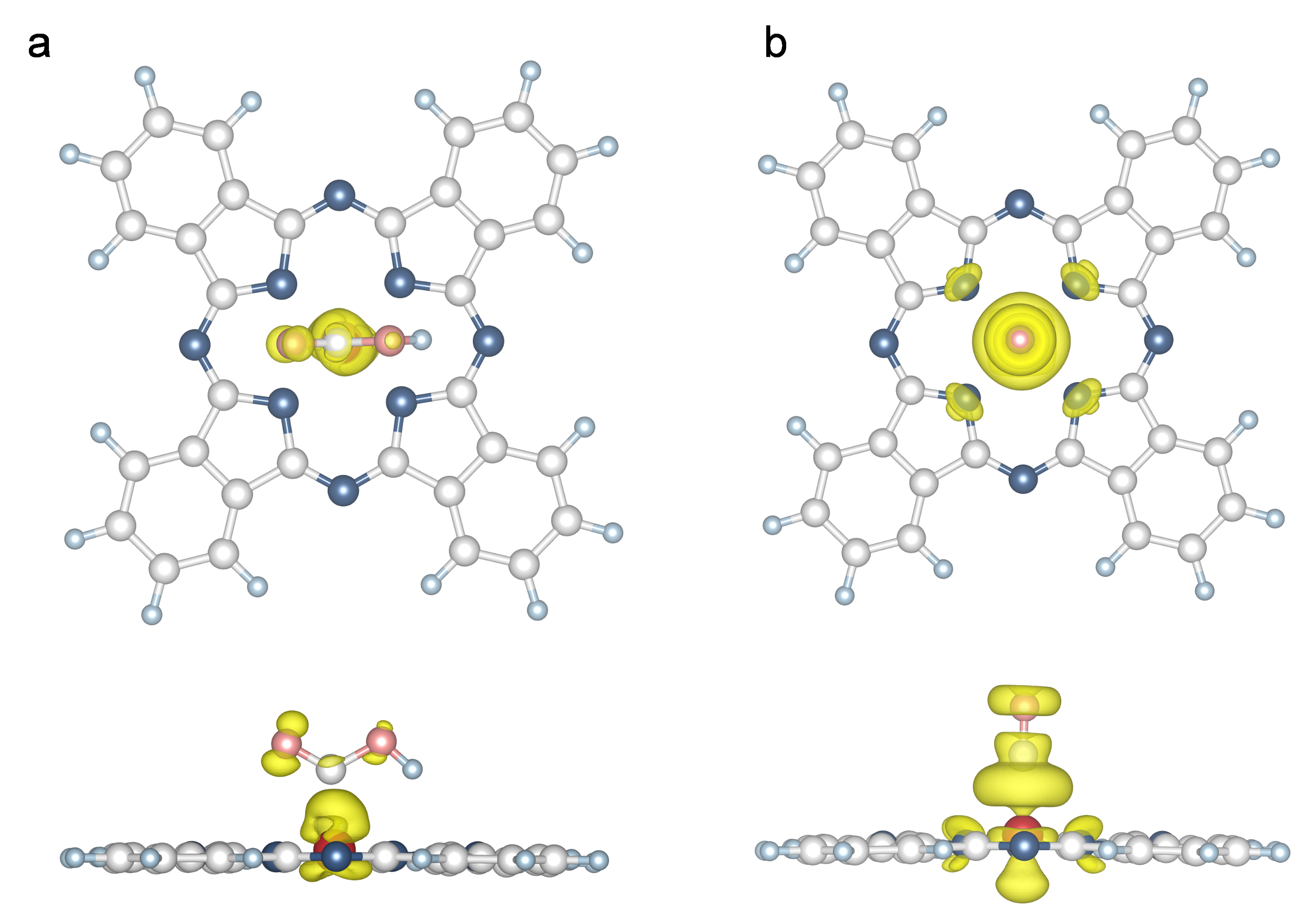


**Supplementary Fig. 37.** Differential charge density of COOH^*^ and CO^*^ on CoPc/GDE during CO_2_RR.


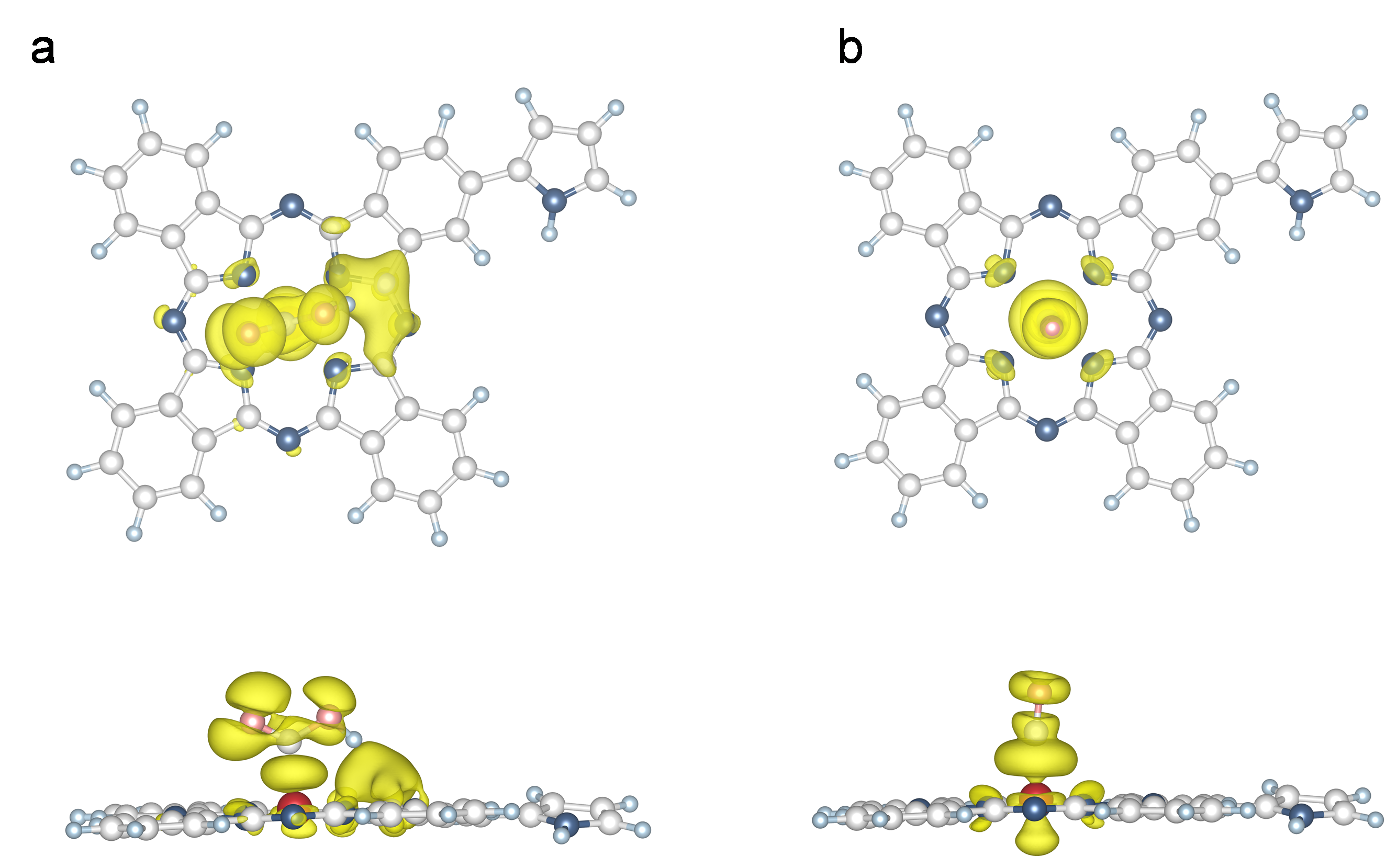


**Supplementary Fig. 38.** Differential charge density of COOH^*^ and CO^*^ on CoPc/ppy/GDE during CO_2_RR.


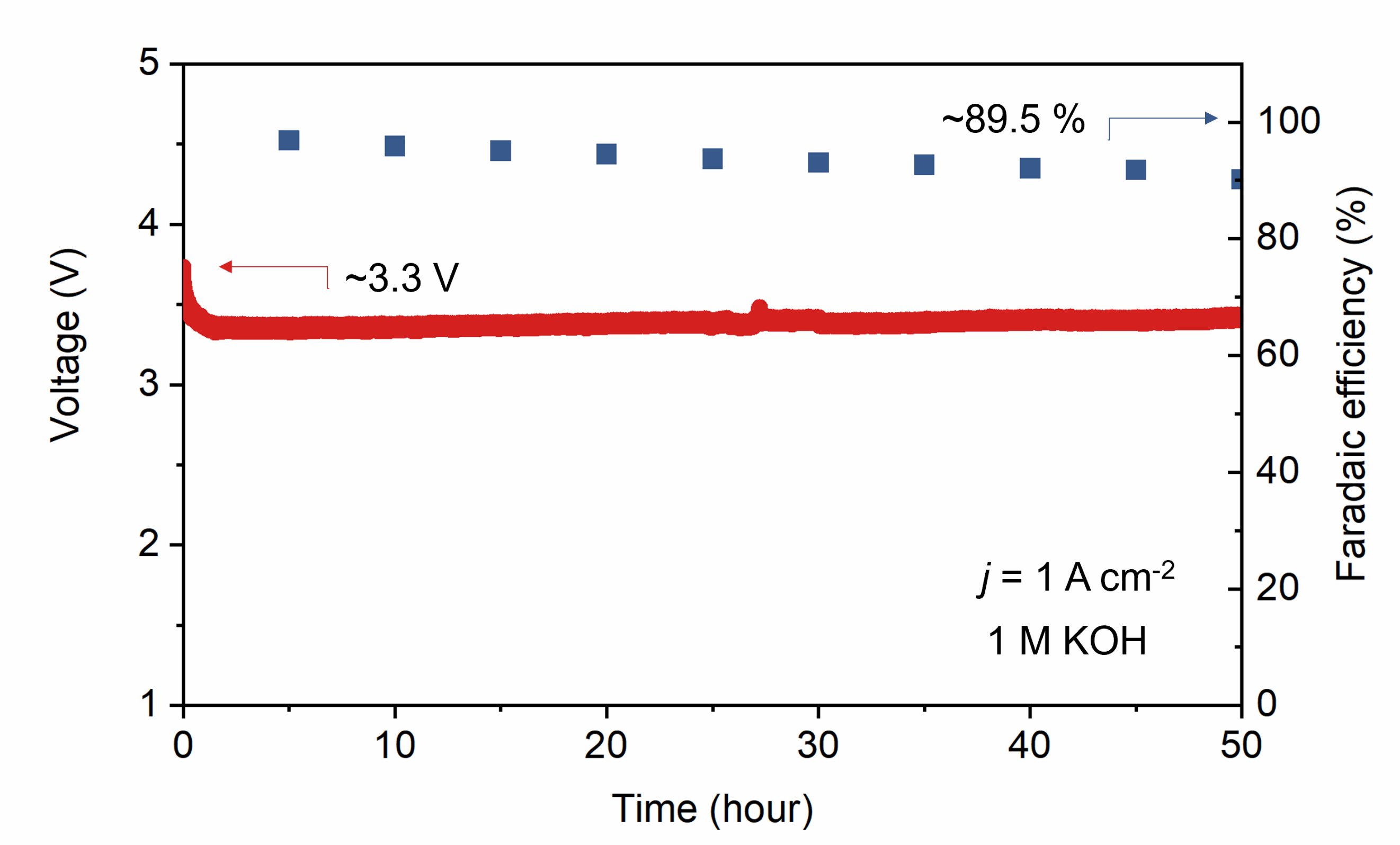


**Supplementary Fig. 39.** Long-term stability test at 1 A cm^−2^ with corresponding Faradaic efficiency of the electrolyzer in 1.0 M KOH. The gas flow rate of MEA is 20 mL/min. All electrochemical measurements were performed at room temperature (23 ± 2℃). There is no iR correction for voltages. Source data are provided as a Source Data file.


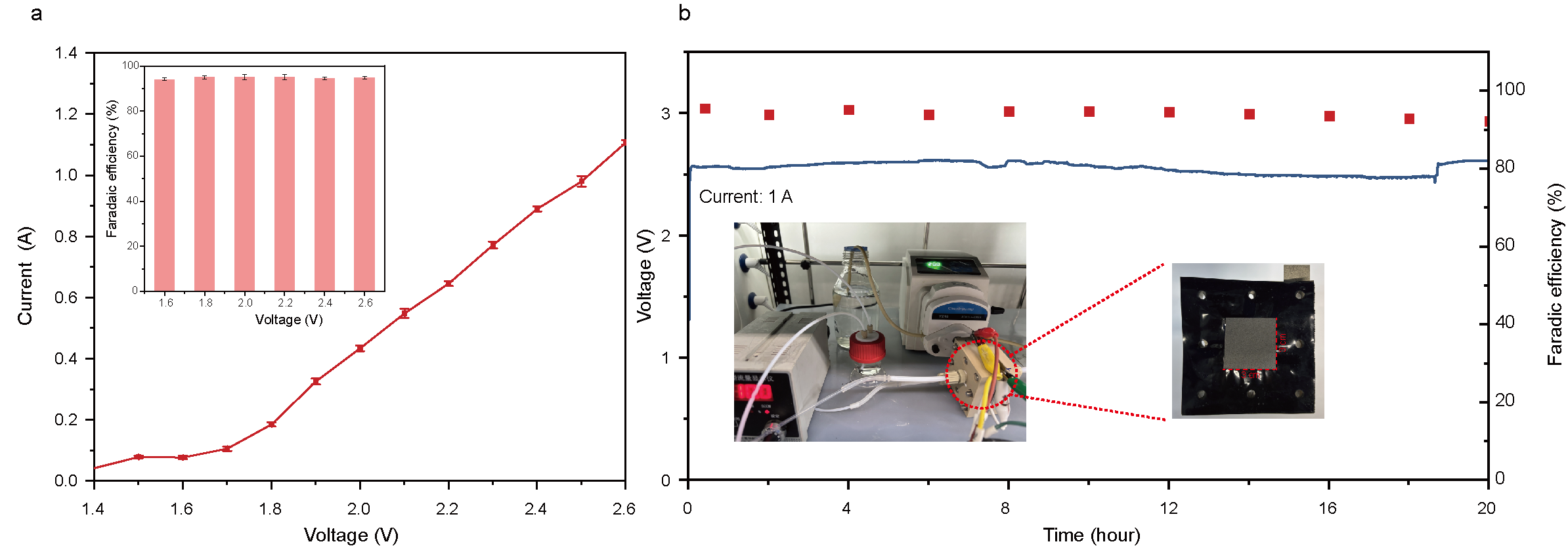


**Supplementary Fig. 40.** **a**, LSV and Faradaic efficiency of CoFe-Ci@GQDs/NF||CoPc/ppy/GDE electrolyzers with electrode area of 9 cm^2^ during CO_2_RR in 1.0 M KOH. **b**, Stability test at 1 A with corresponding Faradaic efficiency of the electrolyzer in 1.0 M KOH. The gas flow rate of MEA is 50 mL/min. All electrochemical measurements were performed at room temperature (23 ± 2℃). There is no iR correction for voltages. Data are presented as mean values ± standard deviation. The standard deviation is obtained based on three independent samples. Source data are provided as a Source Data file.


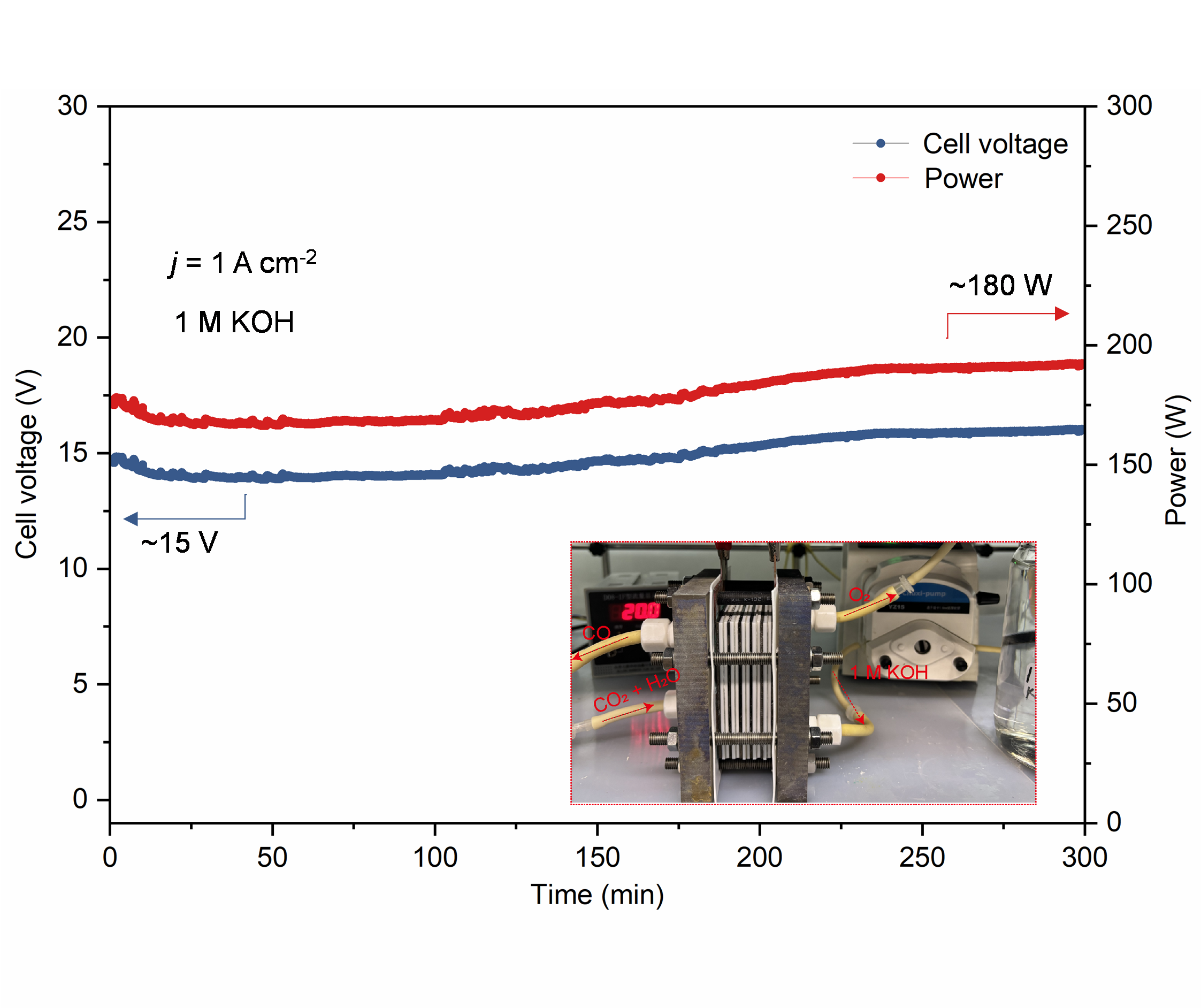


**Supplementary Fig. 41.** Stability and power of the electrolyzer at a current density of 1 A cm^−2^. The gas flow rate of MEA is 100 mL/min. All electrochemical measurements were performed at room temperature (23 ± 2℃). There is no iR correction for voltages. Source data are provided as a Source Data file.

**Supplementary Table 1**. Co content of CoPc/ppy/GDE, CoPc/CNT and CoPc/GDE

| **Catalyst** | **Content (wt. %)** |
| --- | --- |
| CoPc/ppy/GDE | 0.0837% |
| CoPc/CNT-GDE | 6.4097% |
| CoPc/GDE | 0.0046% |

**Supplementary Table 2.** Comparison of the CO_2_-to-CO conversion of different electrocatalysts in H-type cell.

| **Catalysts** | **Electrolyte** | **FE_CO_**  **(%)** | **Potential**  **(V vs. RHE)** | **J**  **(mA cm^−2^)** | **Stability (h)** | | **Ref.** |
| --- | --- | --- | --- | --- | --- | --- | --- |
| CoPc/ppy/GDE | 0.1 M KHCO_3_ | 96 | −0.78 | −40 | 200 | This work | |
| EP-CoP | 0.5 M KHCO_3_ | 95 | −0.62 | −8.5 | 42 | 1 | |
| CoTPP/CNT | 0.5 M KHCO_3_ | 91 | −0.62 | −3.2 | 4 | 2 | |
| FePGF/CFP | 0.1 M KHCO_3_ | 98.7 | −0.54 | −1.68 | 10 | 3 | |
| Co-PMOF | 0.5 M KHCO_3_ | 98.7 | −0.8 | −18.3 | 36 | 4 | |
| STPyP-Co | 0.5 M KHCO_3_ | 96 | −0.62 | −6.6 | 48 | S5 | |
| Fe-PB | 0.5 M KHCO_3_ | 100 | −0.63 | ~−0.6 | 24 | 6 | |
| Co-TTCOF | 0.5 M KHCO_3_ | 91.3 | −0.7 | −2.02 | 40 | 7 | |
| COF-366-(OMe)_2_-Co@CNT | 0.5 M KHCO_3_ | 93.6 | −0.68 | ~−6 | 12 | 8 | |
| COF-367-Co | 0.5 M KHCO_3_ | 91 | −0.55 | −3.3 | 24 | 9 | |
| CoPc-TFPN COF | 0.5 M KHCO_3_ | 99.8 | −0.9 | −14.1 | 60 | 10 | |
| TT-Por(Co)-COF | 0.5 M KHCO_3_ | 91.4 | −0.6 | ~−1.5 | 10 | 11 | |
| CoPc-PI-COF-1 | 0.5 M KHCO_3_ | 90 | −0.7 | −10 | 40 | 12 | |
| MWCNT-Por-COF-Co | 0.5 M KHCO_3_ | 88 | −0.7 | −6 | 50 | 13 | |
| CoPc/CNT | 0.1 M KHCO_3_ | 92 | −0.63 | −10 | 10 | 14 | |
| CoTMAPc@CNT | 0.5 M KHCO_3_ | 95 | −0.62 | ~−12 | 12 | 15 | |
| CoPPc/CNT | 0.5 M NaHCO_3_ | 90 | −0.54 | −12 | 24 | 16 | |
| CoPc-2H2Por | 0.5 M KHCO_3_ | 95 | −0.6 | −5 | 70 | 17 | |

**Supplementary Table 3**. The values derived from EIS Nyquist plot fitting.

|  | Rs | R1 | R2 |
| --- | --- | --- | --- |
| CoPc/ppy/GDE | 9.81 | 4.532 | 38.47 |
| CoPc/GDE | 10.59 | 9.678 | 69.2 |

**Supplementary Table 4.** Comparison of the CO_2_-to-CO conversion of different electrocatalysts in MEA.

| **Catalysts** | **Electrolyte** | **FE_CO_** | **Cell voltage (V)** | **J (mA cm^−2^)** | | **Stability (h)** | **References** |
| --- | --- | --- | --- | --- | --- | --- | --- |
| CoPc/ppy/GDE | 1.0 M KOH | 99% | ~2.8 | 500 | | 120 | This work |
| CoPc/ppy/GDE | 1.0 M KOH | 98% | ~3.2 | 1000 | | 50 | This work |
| Co-CNTs MW | 1.0 M KOH | 98.3% | ~2 | 100 | | 12 | 18 |
| CoPc | 1.0 M KOH | 88% | ~2.52 | 200 | | 6 | 19 |
| MWNT/  PyPBI/Au | 2.0 M KOH | 85% | ~2.25 | 158 | | 8 | 20 |
| Ag/C | 1.0 M KOH | 83% | ~2.75 | 120 | | 0.417 | 21 |
| Ag  nanoparticles | 1.0 M  KOH+0.33 M  Urea | 98% | ~2.16 | 100 | | 10 | 22 |
| Zn_2_P_2_O_7_ | 1.0 M KOH | 93.9% |  | 100 | | 7 | 23 |
| Au_24_ | 0.1 M KOH | 90.0% | ~3 | 100 | | 100 | 24 |
| TC-CoPc/MWCNTs | 0.5 M KHCO_3_ | 97% | ~2.6 | | 50 | 60 | 1 |
| CoPc/Mg(OH)_2_ | 0.1 M KHCO_3_ | 95% | 3.4 | | 100 | 20 | 25 |
| β-CoPc/CP | 1.0 M KOH | 92% | 2 | | 100 | 29.3 | 26 |
| AG-CoPc/MWCNTs | 0.1 M KHCO_3_ | 80% | 3 | | 50 | 12 | 27 |
| MD-CoPc/MWCNTs | 0.1 M KHCO_3_ | 80% | 3.2 | | 50 | 40 | 27 |

References

1. Wang, C. et al. In situ electropolymerized 3D microporous cobalt-porphyrin nanofilm for highly effective molecular electrocatalytic reduction of carbon dioxide. Adv. Mater. **35,** 2303179 (2023).
2. Hu, X. M. et al. Enhanced catalytic activity of cobalt porphyrin in CO_2_ electroreduction upon immobilization on carbon materials. Angew. Chem. Int. Ed. **56**, 6468–6472 (2017).
3. Choi, J. et al. A Porphyrin/graphene framework: A highly efficient and robust electrocatalyst for carbon dioxide reduction. Adv. Energy Mater. **8**, 1801280 (2018).
4. Wang, Y. R. et al. Oriented electron transmission in polyoxometalate-metalloporphyrin organic framework for highly selective electroreduction of CO_2_. Nat Commun. **9**, 4466 (2018).
5. Han, J. Y. et al. Reordering d orbital energies of single-site catalysts for CO_2_ electroreduction. Angew. Chem. Int. Ed. **58**, 12711–12716 (2019).
6. Smith, R. T. et al. Iron porphyrins embedded into a supramolecular porous organic cage for electrochemical CO_2_ reduction in water. Angew. Chem. Int. Ed. **57**, 9684–9688 (2018).
7. Zhu, H. J. et al. Efficient electron transmission in covalent organic framework nanosheets for highly active electrocatalytic carbon dioxide reduction. Nat Commun. **11**, 497 (2020).
8. Lu, Y. et al. Efficient carbon dioxide electroreduction over ultrathin covalent organic framework nanolayers with isolated cobalt porphyrin units. ACS Appl. Mater. Interfaces **12**, 37986 (2020).
9. Lin, S. et al. Covalent organic frameworks comprising cobalt porphyrins for catalytic CO_2_ reduction in water. Science **349**, 1208–1213.
10. Lu, M. et al. Stable dioxin-linked metallophthalocyanine covalent organic frameworks (COFs) as photo-coupled electrocatalysts for CO_2_ reduction. Angew. Chem. Int. Ed. **60**, 4864–4871 (2021).
11. Wu, Q. et al. Construction of donor–acceptor heterojunctions in covalent organic framework for enhanced CO_2_ electroreduction. Small **17**, 2004933 (2021).
12. Han, B. et al. Two-dimensional covalent organic frameworks with cobalt(ii)-phthalocyanine sites for efficient electrocatalytic carbon dioxide reduction. J. Am. Chem. Soc. **143**, 7104–7113 (2021).
13. Dong, H. et al. Covalently anchoring covalent organic framework on carbon nanotubes for highly efficient electrocatalytic CO_2_ reduction. Appl. Catal., B **303**, 120897 (2022).
14. Zhang, X. et al. Highly selective and active CO_2_ reduction electrocatalysts based on cobalt phthalocyanine/carbon nanotube hybrid structures. Nat Commun. **8**, 14675 (2017).
15. Sun, J. J. et al. Building a stable cationic molecule/electrode interface for highly efficient and durable CO_2_ reduction at an industrially relevant current. Energy Environ. Sci. **14**, 483–492 (2021).
16. Han, N. et al. Supported cobalt polyphthalocyanine for high-performance electrocatalytic CO_2_ reduction. Chem **3**, 652–664 (2017).
17. Yuan, J. J. et al. Structural Regulation of coupled phthalocyanine–porphyrin covalent organic frameworks to highly active and selective electrocatalytic CO_2_ reduction. Adv. Mater. **34**, 2203139 (2022).
18. Sun, J. W. et al. Scalable synthesis of coordinatively unsaturated metal-nitrogen sites for large-scale CO_2_ electrolysis. Nat. Commun. **1599**, 4582 (2023).
19. Ren, X. X. et al. Molecular electrocatalysts can mediate fast, selective CO_2_ reduction in a flow cell. Science **365**, 367–369 (2019).
20. Verma, S. et al. Insights into the low overpotential electroreduction of CO_2_ to CO on a supported gold catalyst in an alkaline flow electrolyzer. ACS Energy Lett. **3**, 193–198 (2017).
21. Endrodi, B. et al. Multilayer electrolyzer stack converts carbon dioxide to gas products at high pressure with high efficiency. ACS Energy Lett. **4**, 1770–1777 (2019).
22. Wang, L. et al. Regulating the local charge distribution of Ni active sites for the urea oxidation reaction. Angew. Chem. Int. Ed. **60**, 10577–10582 (2021).
23. Zhang, X. Y. et al. In operando identification of in situ formed metalloid Zinc^δ+^ active sites for highly efficient electrocatalyzed carbon dioxide reduction. Angew. Chem. Int. Ed. **61**, e202202298 (2022).
24. Kulkarni, V. K. et al. N-heterocyclic carbene-stabilized hydrido Au_24_ nanoclusters: Synthesis, structure, and electrocatalytic reduction of CO_2_. J. Am. Chem. Soc. **144**, 9000–9006 (2022).
25. Lyn, F. et al. Pre-activation of CO_2_ at cobalt phthalocyanine-Mg(OH)_2_ interface for enhanced turnover rate. Adv. Funct. Mater. **33**, 2214609 (2023).
26. Wu, X. F. et al. Isolation of highly reactive cobalt phthalocyanine via electrochemical activation for enhanced CO_2_ reduction reaction. Small **19**, e2207037 (2023).
27. Wu, X. F. et al. Molecularly dispersed cobalt phthalocyanine mediates selective and durable CO_2_ reduction in a membrane flow cell. Adv. Funct. Mater. **32**, 2107301 (2021).
