# Supplementary material for: In-situ grafting of cobalt phthalocyanine on gas diffusion electrodes enables ampere-level CO2 reduction
Source: Nat Commun. 2025 Dec 8;17:124. doi: 10.1038/s41467-025-66808-3 (PMC12775133; doi:10.1038/s41467-025-66808-3)
Supplement: Supplementary file 2 — Description of Additional Supplementary Files [file 41467_2025_66808_MOESM2_ESM.pdf]

Description of Additional Supplementary Files

File Name: Supplementary Data 1

Description: The atomic coordinates of the optimized computational models.
